# Pangenome Analysis of *Proteus mirabilis* Reveals Lineage-Specific Antimicrobial Resistance Profiles and Discordant Genotype-Phenotype Correlations

**DOI:** 10.1101/2025.11.21.689858

**Published:** 2025-11-24

**Authors:** Namrata Deka, Aimee L. Brauer, Katherine Connerton, Blake Hanson, Jennifer N. Walker, Chelsie E. Armbruster

## Abstract

Urinary tract infections (UTIs) impose a substantial health care burden with increasing antimicrobial resistance and treatment failure rates. *Proteus mirabilis* is a challenging UTI pathogen due to intrinsic resistances coupled with formation of crystalline biofilms. We combined pangenome analysis, antimicrobial resistance gene (AMR) predication, and antimicrobial susceptibility testing (AST) to determine whether highly multidrug-resistant (MDR) isolates of *P. mirabilis* arise from distinct lineages and explored the clinical utility of multilocus sequence typing (MLST). The pangenome of 1,001 *P. mirabilis* genomes from human urine revealed an open conformation driven by strain diversity and the accessory genome. A total of 213 sequence types (STs) were identified and only 7% had ≥11 genomes, highlighting strain diversity. 93% of the *P. mirabilis* genomes harbored resistance genes for ≥2 antibiotic subclasses, and 25% were predicted to be resistant to >6 subclasses, confirming a high MDR burden. By focusing on the 15 most prevalent STs, we observed that AMR subclasses were largely lineage-specific. However, isolates with very high resistance gene counts (>20) were distributed across multiple STs, indicating that extreme resistance gene accumulation is not restricted to ST. Comprehensive AST of 27 *P. mirabilis* clinical isolates further revealed variable genotype-phenotype concordance, indicating unresolved mechanisms of resistance not captured in current AMR prediction databases. In summary, our study emphasizes the critical need to expand genomic surveillance of *P. mirabilis* to fully understand the complex landscape of AMR in this species and translate these insights into improved diagnostic and therapeutic strategies.

**IMPORTANCE:**

*Proteus mirabilis* is a clinically-challenging cause of urinary tract infections due to multidrug resistance and its ability to form crystalline biofilms that provide further antibiotic protection. In this study, we sought to determine how well sequence typing and antimicrobial resistance gene prediction correlate with laboratory-based antimicrobial susceptibility testing. By analyzing more than 1,000 *P. mirabilis* genomes from human urine samples, we found that some resistance patterns were sequence type-specific. However, the genome structure of this species suggests frequent horizontal gene transfer, and the most highly-resistant strains did not cluster by lineage. Importantly, many isolates that appeared “susceptible” based on their genomes were in fact resistant upon laboratory testing, revealing hidden or uncharacterized resistance mechanisms. These findings show that current gene-based prediction tools can miss clinically relevant resistance, underscoring the need for further study to guide effective treatment of *P. mirabilis* infections.

**Author Contributions:** ND and CEA designed experiments. ND, KC, and ALB conducted MIC experiments, ND and CEA analyzed results. ND performed genome sequencing, pangenome analysis, and AMR detection. BH and JW provided guidance on pangenome analysis, AMR detection, and data visualization. ND visualized the data and prepared the first draft of the manuscript. CEA provided project administration. All authors critically reviewed and revised the manuscript.

## INTRODUCTION

Urinary tract infections (UTIs) are among the most common bacterial infections, with an estimated healthcare cost of >$3 billion in the United States [1-4]. They are a leading cause of antimicrobial prescriptions in both outpatient and inpatient settings, with 50-60% of women experiencing at least one UTI in their lifetime [5]. UTI can be classified as complicated UTI (cUTI) or uncomplicated UTI (uUTIs) depending on the presence and absence of structural or functional abnormalities of the urinary tract, catheterization, or immunocompromised states [5]. Recent epidemiological data estimate approximately 174,000 cases of uUTI and 2.38 million cases of cUTI annually [6, 7]. Despite standard antibiotic therapy, treatment failure rates for UTIs remain prevalent, occurring in 16.7% of cases in adult female outpatients with cUTI, 27% of elderly men, and 15% of elderly women, often leading to recurrence, secondary infection, progression to pyelonephritis, or urosepsis [8, 9].

Among uropathogens, *Proteus mirabilis* is an important Gram-negative bacterium and a major cause of UTIs, contributing to approximately 4.2% of uUTIs and 4.7% of cUTIs [5]. Notably, *P. mirabilis* accounts for up to 45% of catheter-associated UTIs (CAUTIs) and 13-21% of secondary bacteremias, making it a key pathogen in medical device-associated infections [5, 10, 11]. *P. mirabilis* has a strong propensity for biofilm formation and causes encrustation of the catheter by deposition of struvite and apatite crystals, leading to persistent infections and catheter blockage. Struvite and apatite crystals form due to the production of a potent urease enzyme by *P. mirabilis* that increases urine pH, promoting ion precipitation, crystal deposition, and urinary stone formation [10, 12-15]. It can remain in bladder crystalline deposits despite multiple doses of antibiotic treatment, catheter changes, and even periods without catheterization [15-17]. Infection with *P. mirabilis* can become fatal when it progresses to symptomatic CAUTI, acute bacteremia, and urosepsis due to its ability to disseminate from the bladder to other organs [14, 18].

Antimicrobial resistance (AMR) in UTI pathogens is an escalating global concern, with increasing reports of multidrug-resistant (MDR) strains complicating treatment [19]. Treatment failure for *P. mirabilis*-associated UTIs has been linked to MDR, leading to prolonged hospitalization, increased healthcare costs, and higher morbidity rates [15, 19, 20]. Treatment typically involves antibiotics but becomes challenging due to intrinsic and acquired resistance mechanisms, including AmpC β-lactamase production, efflux pumps, and extended-spectrum β-lactamase (ESBL) expression [14, 21-23]. These factors contribute to resistance to first-line UTI antibiotics such as trimethoprim-sulfamethoxazole (TMP-SMX), fluoroquinolones, and β-lactams. The prevalence of ESBL genes like *blaTEM* and *blaCTX-M* further exacerbates resistance, while mobile genetic elements such as SXT/R391 integrative and conjugative elements (ICEs) play a crucial role in the spread of AMR genes in *P. mirabilis* strains [19, 24, 25].

Numerous studies have characterized the model *P. mirabilis* strain HI4320, originally isolated from the female urinary tract, including virulence factors such as adhesins for attachment and biofilm formation, flagella for motility and dissemination, urease enzyme activity leading to renal calculi, and iron-binding siderophores [10, 26]. However, a major gap remains in our understanding of the diversity of strains isolated from clinical urine samples, either during infection or in the presence of a catheter. Multilocus sequence typing (MLST) provides an unambiguous method for characterizing bacterial isolates based on the sequences of internal fragments of typically 6-7 housekeeping genes. An open question in the field is whether specific sequence types (STs) in *P. mirabilis* are associated with the accumulation of similar AMR and virulence genes. While previous studies in other bacterial species suggest that certain lineages correlate with varying levels of resistance and biofilm production, such associations in *P. mirabilis* remain largely unexplored. For example, in methicillin-resistant *Staphylococcus aureus* (MRSA), expression of the *ica* genes—critical for biofilm formation—varies among different STs, with certain STs showing higher *ica* gene expression, particularly in strains isolated from wound and catheter samples [27]. If similar correlations exist in *P. mirabilis*, routine sequence typing of clinical isolates could serve as a valuable tool to predict AMR potential and pathogenicity.

Despite advances in next-generation sequencing (NGS), *P. mirabilis* has not been typed extensively at the genomic level. Several typing schemes have been developed to characterize *P. mirabilis* strains, but no standard scheme is followed for clinical classification. Core genome MLST (cgMLST), a high-resolution genomic method, has identified 205 clonal groups (CGs) linked to severe UTIs and AMR [28]. Some other older methods include proticine production and sensitivity typing, which differentiates strains based on bacteriocin activity, and bacteriophage typing, which classifies strains by susceptibility to phage lysis [29, 30]. Additionally, Dienes typing, based on swarming behavior, provides a simple means of strain differentiation, while serotyping classifies strains based on O-antigen structures, revealing a diverse antigenic profile among clinical isolates [31]. Recent comprehensive genomic studies by Potter *et. al.* and Lian *et. al*. have given insights into some key genomic features of *P.*

*mirabilis* populations and distribution [32, 33]. However, there remains a lack of data on the prevalence of *P. mirabilis* isolates through a single classification system and the link between ST to AMR genes in urine isolates. This gap in knowledge highlights the need to investigate whether specific STs are predictive of resistance patterns or increased virulence within the urinary tract. By focusing on genomes isolated from human urine samples, we aim to gain critical insights into AMR and virulence of *P. mirabilis* in urine to have better indicators for risk of treatment failure and severe disease.

There are numerous ways to predict AMR, including the standardized clinical laboratory practices of testing resistance phenotypes to specific antibiotics, as well as genomic or machine learning methods to predict resistant gene expression [34]. Genomic prediction tools can be useful for quick detection of AMR in clinical practice, and there may be a correlation between AMR gene prediction and phenotypic expression, but the nature of the correlation can vary depending on specific genes and the bacterial species. For instance, *Escherichia coli* frequently harbors resistance genes for β-lactams, sulfonamides, trimethoprim, and chloramphenicol on mobile genetic elements, leading to a stronger correlation between genotype and phenotype [35-37]. In contrast, according to some studies, *Proteus mirabilis* carrying β-lactam and sulfonamide (*sul1* and *sul2*) genes exhibits this correlation less consistently, showing weaker alignment between genomic predictions and observed resistance profiles [35, 38-40].

Tools like AMRFinder and CARD are accurate *in silico* prediction tools; AMRFinder was able to validate the presence of genes pertaining to AMR phenotypes for *Salmonella enterica, Campylobacter spp*., and *Escherichia coli* with 98.4%

consistent predictions [41]. Further, a 99% concordance was found between phenotypic antimicrobial susceptibility testing and *in silico* prediction of AMR genes using whole-genome sequencing (WGS) in 1,321 isolates of *Salmonella enterica* from human infections in Canada [20]. Similarly, a machine-learning approach was used to predict the Minimum Inhibitory Concentrations (MIC) of ciprofloxacin in *Escherichia coli* where MIC was predicted accurately in 65%-93% of the samples within a 2-to-4-fold dilution range [42]. While genetic data provides a promising avenue to predict AMR, it is important to understand the limitations and variability in gene expression and resistance phenotypes in different bacteria. There has been no such correlation established for *P. mirabilis,* and it would be particularly important to examine for urine isolates. Hence, integrating genotypic AMR prediction and phenotypic MIC testing in diverse strains may offer a more comprehensive approach to predicting antimicrobial resistance in this species.

In this study, we conducted a comparative genomic analysis of *P. mirabilis* using 1,001 publicly available human urine isolate sequences to characterize the pangenome, prevalent MLST in clinical isolates, and AMR profiles. Additionally, we compare these findings with 26 clinical isolates collected from patients with long-term indwelling catheters [15, 17]. Given the increasing challenges associated with AMR and persistent infections, we sought to identify predictive factors that correlate genomic data with resistance mechanisms in a clinical setting. By evaluating urine isolates from both community and healthcare settings, we aimed to provide a comprehensive understanding of *P. mirabilis* antimicrobial susceptibility patterns and resistance determinants. We found that *P. mirabilis* isolates from urine exhibit a heterogenous

AMR gene profile that is independent of collection source. However, similar MLSTs clustered together regardless of isolation source and showcased related AMR profiles. Importantly, the intrinsic resistance of *P. mirabilis* to tetracycline and the high incidence of TMP-SMX resistance eliminates the use of two primary UTI therapy agents. This necessitates reliance on more expansive broad-spectrum alternatives, fueling the AMR crisis. Our study also demonstrates that numerous *P. mirabilis* CAUTI isolates that would be classified as antibiotic susceptible by AMR gene prediction were resistant upon phenotypic testing, indicating that additional as-yet unidentified resistance mechanisms may be prevalent in urine isolates of this species and likely to influence treatment efficacy in clinical settings.

## MATERIALS AND METHODS

### Data availability statement

Sequencing data have been deposited in NCBI BioProject PRJNA1367153: *Proteus mirabilis* genome sequences from human urine sources in the United States. All other data are available within the article and its supplemental material.

### Ethics approval

This body of work includes sequencing of *P. mirabilis* strains that were previously isolated from human subjects in two different studies, for which human subject protocols were approved by the University of Buffalo (IRB #STUDY00002526) and Washington University School of Medicine (IRB #201410058).

### Patient population and Sample collection

To investigate whether *P. mirabilis* MLSTs correlate with AMR patterns, we assessed *P. mirabilis* genomes from three separate cohorts: publicly available genomes from NCBI (**Table S1**), nursing home residents with long-term catheters (CEA isolates), and community-dwelling catheterized patients (HUC isolates).

For comparison of *P. mirabilis* genomes, we sought to query all publicly available high-quality genomes of *P. mirabilis* isolates obtained from urine samples in hospital environments in the United States. Using advanced search parameters, publicly available raw genome data were retrieved from the NCBI database. The initial search, conducted in October 2024, yielded 1,337 hits using the terms “*Proteus mirabilis*,”

isolation source "urine," and host "*Homo sapiens*." Filtering the results to include only isolates originating from the "United States" reduced the dataset to 1,152 genomes. The dataset was further refined by only including whole-genome sequencing data. These filters resulted in a final dataset of 1,064 (**Table S1**) genomes, which were subsequently downloaded from NCBI. Following FastQC v0.11.9-Java-11 analysis and adapter trimming using trimmomatic v0.39-Java-11.0.16. [43, 44], 1,026 genomes were retained for downstream analyses. Samples were excluded if they exhibited poor quality, had high GC content, high read duplication rates, or if paired end read files were missing (46 samples). Raw paired end read sequences were assembled using spades v3.15.5 [45]. After genome assembly, we assessed quality and completeness using QUAST (**Table S2**), selecting 1,001 high-quality genomes with >95% completeness and fewer than 1,000 contigs which were annotated with prokka v1.14.5 to facilitate downstream analyses [46, 47].

Isolates designated 101–210 were cultured from urine specimens collected during a prospective cohort study on asymptomatic catheter-associated bacteriuria at two nursing homes in Buffalo, New York, USA, between July 2019 and March 2020 [15]. Inclusion criteria required participants to be 21 years or older at the time of consent and have an indwelling urinary catheter in place for at least 12 months at the start of the study. Study participants underwent an initial enrollment visit, followed by weekly study visits for up to seven months. Most of the study participants were White (79%) and male (79%), an average age of 65 years, and 68% had suprapubic catheters [15]. Participants exhibited a high degree of functional dependence in activities of daily living, with the most common comorbidities including neurogenic bladder (74%), hemiplegia

(42%), diabetes (32%), renal disease (32%), multiple sclerosis (26%), and chronic heart failure (26%) [15]. Urine specimens were cultured on MacConkey agar and Columbia Nutrient Agar, and Gram-negative bacteria were identified to the species level using API-20E test strips (BioMérieux). *P. mirabilis* species designation was further confirmed based on characteristic swarming motility on blood agar plates (Hardy Diagnostics) and whole genome sequencing (**Table S3**).

Additional *P. mirabilis* isolates with designations of “HUC” were obtained from the lab of Scott J. Hultgren, Washington University School of Medicine [17]. These isolates were derived from urine samples collected from community-dwelling catheterized patients during routine catheter removals in the urology clinic as part of their standard care. Most of the participants were male (64%) with an average age of 64 years. Inclusion criteria required participants to be 18 years or older at the time of consent and scheduled for the removal of an indwelling urinary device, including urethral and suprapubic catheters or ureteral stents. The primary indications for catheterization included bladder outlet obstruction (33%), peripheral or central neuropathy (40%), rectovesical or rectourethral fistula (7%), stress urinary incontinence (7%), atonic bladder (11%), and temporary post-operative complications (2%) [17]. Bacterial colonies were distinguished based on morphology, size, and color, and 1–9 single colonies per sample were selected for DNA extraction. *P. mirabilis* isolates were identified via 16S rRNA sequencing and confirmed by whole genome sequencing (**Table S3**).

**Whole Genome Sequencing of Clinical Isolates**

Freezer stocks were streak-plated on MacConkey agar to isolate single colonies of *Proteus mirabilis.* 5 ml of Low Salt Luria Bertani (LSLB; 0.1 g/L NaCl, 10 g/L Tryptone, 5 g/ yeast extract) broth was inoculated with a single colony and incubated for 16 hours at 37°C with shaking. Genomic DNA was extracted using the DNeasy Blood and Tissue Kit (Qiagen, Germantown, Maryland, USA) according to the manufacturer's instructions with specific modifications to ensure high purity of DNA; samples were incubated in proteinase K for 2 hours instead of 10 minutes, and three washes of AW2 buffer were conducted to ensure high-quality DNA free of salts. Samples were sent to SeqCoast Genomics for whole genome sequencing (WGS). All samples were prepared using the Illumina DNA Prep tagmentation kit and IDT For Illumina Unique Dual Indexes. Sequencing was performed on the Illumina NextSeq2000 platform using a 300-cycle flow cell kit to produce 2x150bp paired reads, as previously described [48]. 1-2% PhiX control was spiked into the run to support optimal base calling. The sequencing data has been uploaded in BioProject PRJNA1367153: *Proteus mirabilis* genome sequences from human urine sources in the United States.

Read trimming and run analytics were performed using trimmomatic v0.39.0 [44].

The processed reads were assembled using spades v3.15.5 [45] . The genome assemblies were quality-checked using QUAST v5.2.0 selecting genomes with fewer than 500 contigs (**Table S3**). Genomes that passed quality filtering were annotated using prokka v1.14.5 [47].

**Multi Locus Sequence Typing (MLST) and Phylogenetic trees**

Multilocus sequence typing (MLST) analysis was conducted to identify the sequence types (STs) present in the dataset using mlst software, which incorporates the PubMLST database [49, 50]. The annotated genomes were then fed into roary v3.13.0 using a core threshold cluster of 95%, to identify their pangenome profile and develop a gene presence/absence matrix [51]. The filtered core genome alignment was constructed using FastTree v2.1.11. The resulting newick file was visualized as a mid-rooted phylogenetic tree using the iTOL website and annotated with source, and antibiotic metadata files [52, 53].

**Drug Resistance Gene Prediction**

We used AMRFinderPlus, which uses the Bacterial Antimicrobial Resistance Reference Gene Database to predict AMR genes from the coding sequence of the *P. mirabilis* genomes including the 26 CEA and HUC strains and clinical reference strain HI4320 [34, 41].

**Antibiotic Susceptibility Testing (AST)**

The clinical strains were grown overnight in 5 mL LSLB broth at 37°C with shaking. Antibiotic solutions: Tetracycline HCl (Research Products International (RPI), CAS: 64-75-5), Chloramphenicol (RPI, CAS: 56-75-7), Streptomycin (RPI, CAS: 3810-74-0), Streptothricin (Goldbio, CAS: 96736-11-7), Sulfonamide (sulfamethoxazole, Cayman chemical company, CAS: 723-46-6), Trimethoprim (Cayman chemical company, CAS: 738-70-5), Ampicillin (RPI, CAS: 69-53-4), and Kanamycin (RPI, CAS: 8063-07-8), were prepared in Mueller-Hinton Broth (MHB) at a concentration range encompassing the sensitive-to-resistant spectrum according to Clinical Laboratory Standards Institute (CLSI) guidelines for *Enterobacteriales* [54]. Stock solutions of all the antibiotics were prepared and stored at −20°C, and working dilutions were made fresh from the stock immediately prior to testing.

1ml of the overnight cultures were centrifuged at 1,000g to pellet the cells. The supernatant was removed, and the pellets were resuspended in 0.9% saline to wash away residual LB. The inoculum was adjusted to $4x10^5$ CFU/ml in MHB. One antibiotic with increasing concentrations was tested per 96-well plate, with four technical and duplicate biological replicates. The plates were incubated at 37°C with double-orbital shaking in Biotech Synergy H1, and the $OD_{600}$ was measured every 15 minutes for 20 hours to monitor bacterial growth with $OD_{600}$ threshold set at 0.15.

## RESULTS

To assess the population structure and genetic diversity of *Proteus mirabilis* in the urinary tract, we analyzed 1,001 publicly available genomes isolated from human urine specimens (**Table S1**). The genomes downloaded from NCBI that were used in this analysis were predominantly derived from clinical settings, focusing on medical surveillance and characterization of urinary samples.

Key contributing sources include research from Washington University School of Medicine (WUSM), the University of Pittsburgh Enhanced Detection System for Healthcare-Associated Transmission (EDS-HAT) study, and national antimicrobial surveillance programs such as the Center of Disease Control's Healthcare-Associated Infection Sequencing (CDC HAI-Seq) Gram-Negative Bacteria Study and studies on the human urinary microbiome. Among the genomes, contig counts ranged from 60 to 973, with an average of 258 and a median of 180 contigs. The GC content averaged 38.76%, with a median of 38.69%, making them suitable for downstream analyses (**Table 1 and S2**). While all sequences were confirmed to be derived from urine samples, the clinical context of these specimens—whether they were associated with asymptomatic bacteriuria or infection—is not known.

Phylogenetic analyses (mid-rooted tree) revealed a monophyletic, star-like phylogeny, with a central ancestral node from which several peripheral branches radiate (**Fig 1**), which may indicate recent diversification events. When considering branching, a subset of genomes branched out creating three clades, *clade 1* (964 genomes, dominant), *clade 2* (5 genomes), and *clade 3* (32 genomes) (**Fig S1**). This breakdown is consistent with previously reported intra-species core genome variation in *P. mirabilis*

[32, 33, 55]. For example, Potter *et. al.* studied a cohort of 2,060 *P. mirabilis* genomes collected worldwide from multiple anatomical sources and observed that the essential core genes remained relatively conserved with three divergent sub-species. [33].

**Pan-genomic analysis of *P. mirabilis* reveals a mosaic structure**

To characterize the pangenome of *P. mirabilis* from human urine samples we assessed its structural composition, including core, shell, and cloud gene categories. In the 1,001 genomes analyzed, a total of 41,121 gene families were identified. Of these, the core genome (genes conserved in >99% of the samples analyzed) is small with 1,937 genes indicating a high degree of conservation. Considering that a typical *P. mirabilis* genome harbors roughly 4,600-4,900 coding sequences (CDS), the core genome accounts for approximately 40-45% of the average gene content. This indicates a high conserved genomic backbone with 4.7% of it supporting essential cellular functions, coexisting with an expansive and dynamic accessory genome (**Fig S1**). The shell of the 1,001 genomes contained 1,749 genes (4.08%), and 531 soft core genes shared among a subset of strains that likely contribute to niche-specific adaptations. The majority of genes (37,204 genes, 90%) were classified as cloud genes, making the accessory component the largest in the pangenome. Having a small, stable core and large, variable accessory genome likely represents strain-specific differences driven by horizontal gene transfer.

The pangenome exhibited an open conformation representing a mosaic pattern (**Fig S1**), with the size expanding as additional genomes were analyzed (**Fig 1b**). This expansion reflects the continuous acquisition of novel gene families, highlighting the dynamic nature of the *P. mirabilis* genome and its capacity for adaptation through horizontal gene transfer (**Fig 1c**). The stability of core genes is crucial for the consistent expression of virulence factors, while the expanding accessory genes may help adapt to polymicrobial environments and antibiotic pressures. These findings indicate the genetic plasticity of *P. mirabilis* isolated from urine and provide a foundation for future studies on its pathogenic potential and AMR mechanisms in the urinary tract.

**Publicly available *P. mirabilis* genomes comprise 213 defined and 118 undefined MLSTs**

The specific genes used in MLST of *P. mirabilis* are a combination of housekeeping genes *atpA, dnaJ, mdh, pyrC, recA,* and *rpoD* [49, 50]. We identified a total of 213 defined STs and approximately 118 novel or undefined genomes, reflecting the diversity of *P*. *mirabilis* human urine isolates within the dataset (**Fig 1**). The untyped MLST have been submitted to the PubMLST database to update the schema in *P. mirabilis*.

A detailed analysis of ST prevalence within the dataset revealed that certain STs were more frequently observed. The distribution of MLSTs across the dataset was highly skewed (**Fig 2a, Sup Table S4 and S5**), with 46.26% of the STs having only one representative genome in the dataset while ~7% of the STs had ≥11 genomes (**Fig 2a**). Many genomes in the same ST came from the same study/location but it was not the rule as the top 15 overrepresented ST's show mixed isolation sources (**Fig 1a**). The uneven ST distribution is further visualized in a bar chart of the top 20 MLSTs, highlighting the dominance of a few clonal lineages across sampled populations (**Fig**

**2b**). ST96 was the most abundant (49 genomes; 4.90%), followed by ST185 (42 genomes; 4.20%), ST99 (38 genomes; 3.80%), ST135 (38 genomes; 3.80%), and ST97 (37 genomes; 3.70%). Notably, genomes from each of these STs clustered together in distinct lineages, even if isolated from different geographical locations and reported in different studies (**Fig 1a**). This pattern supports the core genome phylogenetic tree clustering and also that accessory gene variation in the whole genome in *P. mirabilis* are closely linked to clonal lineages, meaning that the non-essential adaptive genes of *P. mirabilis* are not random but are co-related to its evolutionary history linking multiple sources [33].

**Phylogenetic analysis reveals shared lineages and novel variants across clinical *P. mirabilis* isolates**

To begin further examining the clinical relevance of *P. mirabilis* MLSTs, we next sequenced and analyzed a collection of 25 clinical isolates from two distinct cohorts (CEA isolates collected from the urine of nursing home residents with long-term indwelling catheters, and HUC isolates collected from the urine of catheterized community-dwelling individuals during routine catheter changes), as well as the reference strain HI4320 that was previously isolated from the urine of a catheterized nursing home resident (**Fig 3a**). The analysis revealed a population structure forming two primary clades, termed *clade 1* (23 isolates*)* and *clade 2* (3 isolates) (**Fig 3b**). Lack of the third clade identified in the 1,001 genomes was expected considering its low frequency (0.5%) and the small sample size of the clinical isolate collection. Phylogenetic clustering was in accordance with the core genome and gene content irrespective of the clinical origin (HUC vs CEA, **Fig 3a**). Across the 26 isolates, we identified 15 distinct STs; 10 STs were identified in the HUC cohort, 4 in the CEA cohort (includes a novel ST), and 1 represented solely by HI4320. Six of the 10 overrepresented STs in the NCBI genome dataset were also represented among the clinical isolates; namely STs 96, 97, 135, 145, 146, and 185. ST185 was observed in both the CEA and HUC cohorts (H120-04 and 102-00, 206-16), demonstrating genetic overlap across different geographic locations. The two most prevalent STs in the NCBI dataset were also the most prevalent in the clinical isolates: ST185 (3 isolates, 12%) and ST96 (3 isolates, 12%). This concordance with the NCBI dataset suggests these lineages are not only geographically widespread but also clinically prevalent, likely due to conserved traits (e.g., AMR, virulence, or niche adaptation) that enhance their transmission and success across diverse environments. Their presence also confirms that our clinical cohort captured high-impact, epidemiologically relevant STs rather than rare or sporadically enriched variants even with a limited sample size.

Phylogenetic analyses demonstrated moderate genetic diversity overall, with branch lengths suggesting varied degrees of relatedness. The pangenome of the isolates further highlights the heterogenicity within the clinical isolates (**Fig. 3b**), supporting the mosaic lineage of these isolates. Three isolates (H94-00, H81-01, H105-06) exhibited genetic divergence from the rest of the *P. mirabilis* strains (clade 2), possibly due to horizontal gene transfer or independent evolution. The full summary of isolated sources and STs are provided in **Table 2 and Table S3.**

## ***Proteus mirabilis* urine isolates possess resistance factors to multiple subclasses of antibiotics**

Understanding AMR profiles in *P. mirabilis* is crucial to understand its ability to resist commonly used antibiotics including β-lactams, aminoglycosides, sulfonamides, and trimethoprim, since AMR complicates treatment strategies during UTI and contributes to the global public health burden. Analyzing the AMR genes in publicly available *P. mirabilis* genomes can also provide insights into the dissemination of resistance determinants, guiding surveillance and control measures. We first examined the AMR profiles of the 1,001 publicly available *P. mirabilis* genomes*.* A total of 24 antibiotic resistance subclasses were identified, reflecting the diverse mechanisms by which this pathogen can evade antibiotics. The most prevalent genes were those conferring resistance to tetracycline and chloramphenicol, observed in nearly all genomes (**Table S6)**.

*P. mirabilis* is known to have intrinsic resistance to tetracycline, so it was not surprising that tetracycline resistance genes were detected in 991 (99.0%) of the 1,001 genomes, with *tetJ* specifically encoded by 985 of those 991 (99.5%) (**Table 3**). Several other tetracycline resistance determinants, including *tetA, tetB, tetC*, and *tetD*, were detected at lower frequencies. Interestingly, 934/991 (94.2%) of the genomes carried only a single *tet* gene, while 48 (4.8%) carried 2, and 9 (0.9%) carried 3 *tet* genes (**Fig 4a**). In cases of multiple tetracycline resistance determinants, the genes may be functionally redundant or may have been acquired during co-selection of other antibiotics. Notably, a small number of genomes (11, 1.1%) had no detectable tetracycline resistance genes. This could represent a true absence of the genes, or it could be a result of fragmented assemblies or sequence dissimilarity not found in the AMR prediction database.

Chloramphenicol resistance was detected in 952 (95%) of the genomes, with 748 harboring the *catA* gene. Additionally, 69 genomes harbored both *catA* and *catA1,* 43 harbored *catA* and *floR,* and the presence of all three genes was seen in 15 genomes (**Fig 4b, Table S6**). These findings highlight the prevalence of phenicol resistance in *P. mirabilis* genomes despite limited use of chloramphenicol in modern clinical settings, suggesting that selective pressures from other environments or co-selection with other resistance genes may play a role.

Resistance to aminoglycosides, a critical class of antibiotics for treating severe Gram-negative infections, was predicted in 716 (71%) of the genomes. Notably, we observed streptomycin resistance genes in 352 genomes, suggesting the persistence of resistance despite limited clinical use (**Table 3**). The presence of multiple aminoglycoside resistance determinants indicates that *P. mirabilis* maintains a robust arsenal against this class of antimicrobials, including amikacin, gentamycin, kanamycin, streptomycin and tobramycin.

Resistance to trimethoprim, mediated by *dfrA* alleles, was detected in 290 (29%) genomes. The most prevalent allele was *dfrA1* (n=248), followed by *dfrA17* (n=44), *dfrA32* (n=17), *dfrA5* (n=12), *dfrA19* (n=7), *dfrA14* (n=7), *dfrA7* and *dfrA16* (n=4), *dfr12* and *dfrA27* (n=2), and *dfrA10, dfrA42*, and *dfrA46* (n=1). 235 (81.02%) genomes had a single *dfrA* gene, while 50 (17.2%) carried 2, and 5 (1.72%) carried 3 *dfrA* genes (**Fig 4c**). The diversity of *dfrA* genes highlights the adaptive potential of *P. mirabilis* to acquire and maintain resistance determinants. Resistance to sulfonamides was similarly predicted in 286 (28%) genomes, with *sul1* detected in 180, *sul2* in 187, and 81 genomes harboring both *sul1* and *sul2* genes (**Fig 4d, Table S6**). Importantly, 223 (22%) genomes were predicted to encode resistance determinants for both trimethoprim and sulfonamide (**Fig 4e**). Since trimethoprim and sulfamethoxazole are often used in combination, the co-occurrence of these genes, potentially mediated by mobile genetic elements, would likely complicate efficacy of combined treatment. Additionally, we detected 185 genomes (18%) with Extended Spectrum β-Lactamase (ESBL)-associated genes. Of these, 148 harbored the *blaTEM-1* gene, expected to confer resistance to ampicillin (**Table 3**).

Finally, the number of resistance subclasses detected per *P. mirabilis* genome ranged from 1 to 24, with an average of 4.61 AMR subclasses per isolate. A total of 71 genomes (7%) exhibited resistance to only a single antibiotic subclass, most commonly tetracycline. In contrast, 931 (93%) had resistance to two or more subclasses, and a substantial subset of 246 genomes (25%) harbored more than six antibiotic subclasses, indicating a high prevalence of multidrug resistance in this sample set. 176 genomes (18%) had at least one resistance determinant from all five of the above resistance gene groups, while 56 (6%) had combinations of tetracycline, chloramphenicol, and β-lactam resistance genes. Additionally, phylogenetic analyses (**Fig 1a**) determined that certain clusters consistently had resistance genes to nearly all antibiotic classes shown, while others exhibited a complete absence of resistance genes, independent of study source.

**Antibiotic resistance subclasses have a common distribution within lineages**

To evaluate the relationship between ST and AMR burden in *P. mirabilis*, we quantified the number of predicted AMR subclasses per genome and summarized these across 215 distinct STs. Analysis of AMR gene burden across the STs revealed significant lineage-associated differences in resistome size. Several STs exhibited AMR subclass counts substantially exceeding the dataset-wide mean of 4.4 subclasses per genome. Focusing on the STs with ≥3 genomes, ST431, ST425, ST485, ST450, ST421, ST284, ST269, ST 417, and ST 335 demonstrated the highest mean resistance subclass burdens, each surpassing twice the overall mean (**Fig 5a**). For example, ST485 maintained a high AMR burden (subclass range:11-12 subclasses) with minimal intra-ST variability, suggesting a clonally stable and highly resistant lineage. In contrast, ST431 (range: 9-20 subclasses), ST425 (range: 5-24 subclasses) and ST417 (range: 4-15 subclasses) exhibited broad resistance distributions, indicating greater intra-lineage heterogeneity. This diversity may reflect horizontal gene acquisition or ongoing divergent evolutionary trajectories and underscores the complex dynamics of AMR dissemination within clonal backgrounds.

At the population level, the geometric mean resistome size was 3.6 genes, reflecting the typical genome. However, the arithmetic mean was higher at 5.5 genes due to a small subset of highly resistant genomes. 93.8% of our dataset harbored resistance genes for □10 subclasses and 99.8% of our dataset had □20 subclasses, with the 80$^{th}$ percentile at ~6 subclasses (**Fig 5b**). More than 450 genomes had approximately 5 resistance genes, while 35 exhibited elevated AMR gene counts that extend beyond 20 resistance genes; 4 genomes had 30 gene counts, and 1 extreme case had 36 gene counts that skewed the arithmetic mean. Together, these data indicate that the majority of *P. mirabilis* genomes from this collection encode low to moderate numbers of resistance genes, with only a small fraction exhibiting very high AMR gene carriage.

To further explore AMR-ST associations, we analyzed the distribution of resistance subclasses and number of genes across the 10 most prevalent STs where notable inter-lineage variation was observed (**Fig 5c, 5d**). ST96 (n=49 isolates), the most prevalent lineage, exhibited the highest frequency and diversity of resistance genes (range:1-17, mean: 3.7). These genes conferred resistance to aminoglycosides (e.g., amikacin, tobramycin, apramycin, gentamicin), macrolides (azithromycin), streptogramins, and fosfomycin, in addition to tetracycline and chloramphenicol. 14 genomes from ST96 exhibited resistance to >6 antibiotic subclasses, highlighting extensive genetic adaptation, and this was also observed in 7 genomes from ST97 (n= 37 genomes, range: 1-16, mean: 3.27). Similarly, ST185 (n=42 genomes, range: 1-10, mean: 3.78), ST99 (n=38 genomes, range: 1-12, mean: 3.36) and ST135 (n= 38 genomes, range:1-8, mean: 3.05) frequently displayed resistance to >4 subclasses (**Fig 5c, 5d**). Most genomes carried modest resistomes, with those possessing more than 18 genes being uncommon (80$^{th}$ percentile); 93.5 **%** of genomes had fewer than 30 resistance genes, and 98.3 **%** had 50 or fewer genes (**Fig. 5e**). The AMR burden was dispersed across multiple STs rather than confined to a single lineage. Although most lineages carried few resistance gene, ST 96, ST135 and 185 exhibited higher resistance burden overall and may be linked to more extensive AMR profiles both in the urine genomes and our clinical isolates. A notable conservation of AMR profiles was observed within ST, irrespective of their source of isolation. Genomes in STs 97, 185,

495, and 279 –even though isolated in different studies— had similar resistance profiles *within the same ST* (**Fig 1a**). Similarly, ST135 exhibited similar AMR profiles acquiring all important subclasses highlighted in **Fig 1a**.

In contrast, ST145 (n=34 genomes, range: 1-8, mean: 2.41), ST33 (n=32 genomes, range: 1-9, mean 2.81), ST93 (n=29 genomes, range: 1-8, mean: 3.41) and ST 202 (n=28 genomes, range: 1-10, mean: 3.00) typically harbored resistance to just 2-3 subclasses with a few outliers (**Fig 5c, 5d**). Again, within STs 92, 93, 133, 145, 196, and 202 we observe only sporadic accumulation of AMR genes, a pattern that was consistent across all genomes within these ST's (**Fig 1a**). Collectively, these findings highlight the varied and ST-specific resistance gene profiles found in *P. mirabilis* urine isolates. Some STs exhibit consistently high resistance burdens suggesting a shared capability to acquire or retain the same genes, while others show significant variability, indicating that a combination of clonal expansion and horizontal gene transfer likely play an important role in the spread of AMR.

While some resistome subclass profiles correspond to ST, gene composition was not always determined by ST. Ordination analysis using Jaccard distances show mixing of the ST (**Fig. S2a**). Some clustering is seen for ST185 (red circle) and ST135 (green circle). However, some subclasses as well as genes did not co-relate (marked with red and green arrows for ST185 and ST135) with the genomes captured at the time of isolation. Highlighting the top 3 most prevalent STs, we observe a 95% covariance ellipse (**Fig S2b**) without corresponding to specific AMR gene compositions. Additionally, a comparison of Jaccard distances of the resistomes within ST was similar to the differences between different STs (**Sup S2c**), probably driven by the skewed sampling size. However, we see a small number of STs (ST201, ST114, and ST130 in PCoA and ST201, ST279, and ST93 in NMDS) that showcase compact clusters in the top 20 genomes (**Fig S2d and S2e**). This may suggest that common resistome subclasses (ex. Tetracycline, chloramphenicol, aminoglycosides, carbapenemes, penicillins, cephalosporins, TMP, SMX) are ST-specific, but some less common antibiotic subclasses (ex. erythromycin, clindamycin) are spread across STs depicting a dynamic horizontal gene transfer network.

**The distribution of resistance gene burden reveals reservoir-specific selection**

Comparing the 26 clinical isolates and HI4320 against the 1,001 NCBI genomes revealed reservoir-specific selection pressures. In total, resistance genes representing 14 different antibiotic subclasses were identified in the clinical isolates compared to 24 from the NCBI genomes. The 26 clinical isolates exhibited substantial variation in resistance gene burden, ranging from 1 gene (minimum, observed in ST765 and ST591 isolates carrying only a tetracycline resistance determinant) to 15 genes (maximum, in ST135 isolate H115-00), with an average of 3.8 resistance genes per isolate. This diversity included resistance to tetracycline, chloramphenicol, streptomycin, trimethoprim, kanamycin, erythromycin, sulfonamide, β-lactams, and other subclasses (**Table 3 and Table S8**). Tetracycline resistance gene carriage was nearly ubiquitous in both datasets, and the majority of genomes from both datasets also harbored genes for chloramphenicol resistance, demonstrating a strong correlation despite the small size of the clinical dataset. Tetracycline resistance approached fixation; *tetJ* was present in all isolates predicted to be tetracycline resistant from both datasets, and co-occurrence of

*tetJ* with *tetA* (8%, 2/26 clinical) and *tetC* (4%, 1/26 clinical) was also observed. Similarly, chloramphenicol resistance genes demonstrated strong cross-reservoir conservation, with *catA* (type-4) detected in 89% (22/26) of clinical isolates compared to 95% of NCBI genomes, and 23% of clinical isolates (5/22) co-harbored *catA1* while 11% (3/26) harbored no chloramphenicol resistance genes (**Table S8**). Strikingly, one clinical strain (H115-05) exhibited a rare combination of *floR* + *catB3*; *floR* was seen at a frequency of 1.2% in the 1,001 genomes while *catB3* was seen at a frequency of 0.34% (**Fig. 4b**).

Fewer of the clinical isolates had resistance genes for cephalosporins or quaternary ammonium in comparison to the NCBI dataset (**Table 3**). However, clinical isolates showed enrichment of resistance genes under UTI treatment pressure including TMP-SMX (22%, *sul1/sul2*; 37%, *dfr*) and aminoglycosides (streptomycin (37%, most common: *aadA1*), streptothricin (37%, *sat2*), and kanamycin (26%, *aph(3')-Ia*)) (**Table 3**). In terms of extended β-lactam ecology, clinical isolates predominantly carried *blaTEM-1* (18%) while NCBI genomes exhibited broader cephalosporin resistance genes (**Table 3, Table S6**). These patterns demonstrate how reservoir-specific selection pressures like clinical antibiotic exposure may shape distinct resistance landscapes from a conserved genetic backbone.

**Experimental AMR phenotyping of *P. mirabilis* clinical isolates reveals variable genotype-phenotype concordance**

AMRFinderPlus reports gene presence/absence and point mutation but does not infer phenotypic resistance due to factors including gene expression and regulation.

Thus, many of the resistance genes detected by established computational databases may not be relevant for clinical management or antimicrobial surveillance. To determine concordance between *in-silico* AMR predication and phenotypic resistance in *P. mirabilis*, we determined the AMR of *P. mirabilis* clinical isolates by standardized Antimicrobial Susceptibility Tests (AST). We tested growth kinetics of strains with specific resistance genes at 15-minuite intervals over a 20-hour time course to evaluate the minimum inhibitory concentrations as well as more nuanced dose-dependent effects on the growth rates and doubling times of the *P. mirabilis* strains (**Fig 6 and Fig 7**). Antibiotic concentrations were selected based on the Clinical and Laboratory Standards Institute (CLSI) guidelines for *Enterobacterales* and were designed to span the thresholds for sensitive (lowest) to resistant (highest). The genotypic and phenotypic resistance profiles are summarized in **Table S7 and S9.**

*Tetracycline, Kanamycin, Streptomycin, Streptothricin, and Ampicillin*

For tetracycline, 100% of clinical isolates (26/26) exhibited resistance with MIC values >20 µg/mL, which is consistent with the presence of *tetJ*. Growth kinetics demonstrated uniformly robust growth across all concentrations (**Fig 7a**). Thus, a perfect genotype-phenotype concordance was observed. Carriage of any additional *tet* genes (*tetA* or *tetC*) did not alter MIC or growth kinetics within the tested concentration range.

Kanamycin resistance genes (*aac(6')-Ib-cr5, aph(3')-Ia*) were detected in 7/26 clinical isolates and genotype-phenotype concordance was again observed. All 7 isolates encoding the resistance genes had MIC >34 ug/mL, while 102-24, 106-15, H81-

01, H94-04, and HI4320 were predicted to lack all kanamycin resistance determinants and were susceptible, but some exhibited MICs ranging from 14 – 20.5 ug/ml (**Fig 7b**).

Different combinations of streptomycin resistance genes (*aadA1, aph(6)-Id, aph(3”)-Ib, aadA5, aadA2*) were observed in 10/26 clinical isolates (**Fig 6a, Table S8**), and all provided resistance to streptomycin when tested in our AST assay (**Fig 7c**). Unexpectedly, resistance was also observed in 102-24, 106-15, H81-01, H94-00, and reference strain HI4320 which lacks canonical streptomycin resistance genes, suggesting the presence of an alternative resistance mechanism. However, these strains all exhibited a dose-dependent decrease in growth when incubated with streptomycin. It is also notable that clinical isolate 210-04 (1 predicted gene, *aadA1*) exhibited a similar dose-dependent decrease in growth even though other strains (206-05, H97-00, and H97-11) with a singular *aadA1* gene with 100% amino acid sequence similarity to that of 210-04 did not show dose dependency. It is possible that regulation of *aadA1* differs in 210-04, leading to a less efficient or inducible mechanism of resistance compared to the other isolates.

An ampicillin resistance gene (*blaTEM*) was identified in 5/26 clinical isolates and all exhibited MICs >34 ug/mL. The resistance phenotype was also observed in HI4320, which lacks any known β-lactamase genes. However, strains that did not encode *BlaTEM*, including HI4320, exhibited dose-dependent growth inhibition with increasing concentrations of ampicillin, despite displaying resistance at CLSI breakpoints (**Fig 7d**).

Streptothricin resistance gene *sat2* was observed in 10/26 clinical isolates (**Fig 6a, S8**), yet the AST assay revealed a mix of intermediate to resistant phenotypes with MICs ranging from 8 to >22 ug/mL (CLSI breakpoints are ≤8 for susceptible and ≥16 for resistant). Since streptothricin resistance is conferred by *sat1, sat2*, and *sat4* genes, the presence of only one gene may confer only partial resistance. The genotypically-positive isolates 102-00, 206-16 H97-11, H115-00, H115029, H120-00, and H120-04 exhibited dose-dependent decreases in growth with increasing streptothricin concentrations. However, other genotypically positive strains 206-05 and 210-04 were sensitive (**Fig 7e**). HI4320 exhibited robust growth regardless of streptothricin concentration, yet this strain does not encode any known streptothricin resistance genes (**Fig 7eF**). This discordance suggests that HI4320 may possess a novel, non-canonical and potentially constitutive mechanism of high-level streptothricin resistance.

*Sulfonamide and Trimethoprim*

Sulfonamide resistance genes (*sul1* and *sul2)* were both observed in the clinical isolates, with *sul1* present in 3 isolates (H115-0, H115-05, H120-00), *sul2* in 5 isolates (102-00, 206-16, H120-04, H115-00, H115-05), and both genes co-occurring in 2 isolates (H115-00, H115-05) (**Table S7**). AST with sulfamethoxazole revealed high level resistance (MIC >514 ug/ml) in all isolates, irrespective of *sul* gene carriage (single or double), indicating that the presence of an additional *sul* gene did not further elevate resistance. Unexpectedly, even strains that did not encode *sul* (102-24, 106-15, 210-04, H81-01, H94-00, H97-11, and HI4320) had MICs >514 ug/ml, but partial growth inhibition was clearly present even at the lowest tested concentration (250 ug/ml, **Fig. 8a**). The uniform MIC >514 µg/mL across all strains implies clinical resistance per CLSI guidelines, as it far exceeds the susceptible breakpoint (typically ≤38 µg/mL). However, the partial growth inhibition of strains that do not encode *sul* highlights the complexity of interpreting MICs near the resistance threshold. These data suggest that, while canonical *sul*-mediated resistance mechanisms were absent in these isolates, non-genetic or alternative pathways (e.g. efflux pumps, metabolic adaptations) may contribute to the observed resistance (above threshold) phenotype.

Trimethoprim resistance genes were also detected in 10 clinical isolates, with MICs of >15 µg/mL (exceeding clinical breakpoints) regardless of which resistance gene (*dfrA1*, *dfrA17*, or *dfrA32*) was present (**Fig 8b)**. However, one isolate (H97-00) harboring *dfrA1* exhibited dose-dependent growth inhibition, contrasting with other strains harboring *dfrA1* with 100% amino acid similarity that showed no dose-dependency. This implies strain-specific phenotypic variability (e.g., differential gene expression, compensatory mutations) despite shared genetic determinants. The *dfrA*-negative reference strain H81-01 also displayed full resistance, while strain HI4320 that did not carry the *dfrA* gene or gene mutation displayed intermediate resistance and mirrored the dose-dependent growth inhibition of strain H97-00. These observations support the existence of non-*dfrA* resistance mechanisms in HI4320 and H81-01, such as altered folate metabolism or reduced drug uptake. The discordance between genetic markers and phenotypic outcomes also underscores a key challenge: genetic testing alone cannot always predict antibiotic response. However, *dfr* negative strains 102-24, 106-15, and H94-00 were sensitive to trimethoprim, in concordance with the genotype.

Since the genotype-negative strains were all phenotypically resistant to sulfamethoxazole (SMX), albeit with reduced growth rate, we further tested the impact of combinatory treatment (trimethoprim-sulfamethoxazole, TMP-SMX) on *P. mirabilis* growth. This is an important parameter since, historically, TMP-SMX was the first-line empirical treatment for acute uncomplicated UTIs. In the strains exhibiting 100% genotype-phenotype concordance for both SMX and TMP resistance (i.e. true resistant genotypes correlated with resistant phenotypes), all isolates (102-00, 206-16, H115-00, H115-05, H120-00, and H120-04), exhibited complete resistance to the combinatory drug without any dose-dependent decreases in growth (**Fig 8c**). In contrast, strains such as 206-05, 210-04, and H97-11 that were genotypically SMX susceptible TMP resistant but phenotypically resistant to both antibiotics when used alone exhibited a dose-dependent resistance phenotype when treated with TMP-SMX. Additionally, strains that were genotypically predicted to be sensitive to both antibiotics but were phenotypically resistant demonstrated intermediate to resistant phenotypes for combination treatment: H81-01 displayed an MIC of 8/152 ug/mL (resistant), while 102-24 displayed an MIC >2/38 ug/mL (intermediate). This is clinically concerning as growth was observed at breakpoint concentrations. Other discordant strains that had lower growth yields under single SMX exposure (106-15, H94-00, H97-00, HI4320) were found to be sensitive to the TMP-SMX combinations (**Fig 8c**).

*Chloramphenicol*

Chloramphenicol resistance displayed genotype-phenotype concordance, with all isolates possessing a *catA* gene exhibiting resistance and those lacking *cat* genes exhibiting susceptibility (**Fig 9a**). MIC values ranged from 10-14 ug/ml in isolates lacking a *cat* gene but were >26 ug/ml in isolates with *catA* alone and >34 ug/ml in isolates harboring both *catA* and *catA1* (**Table S9**). Interestingly, the majority of isolates with a single *catA* gene (16/17) exhibited dose-dependent growth inhibition (**Fig 9**), while isolates possessing both *catA* and *catA1* demonstrated hyper-resistance (hR) with no dose dependency (**Fig 9**). The exception was HI4320, which only encodes *catA* but exhibited robust growth at all but the highest three concentrations of chloramphenicol. No strains were predicted to have *catA1* alone, so we were not able to determine whether *catA1* provides a greater level of resistance than *catA,* but our data suggests that the presence of two *catA* genes enhances functional resistance.

We next compared the sequences of the *catA* and *catA1* genes in our *P. mirabilis* clinical isolates that harbored both genes as *catA1* has not previously been reported in *P. mirabilis*. The two genes share 76% amino acid sequence identity (with representative strain 102-00; **Sup Fig 3a**), and *catA* from strain 102-00 showed 100% identity to the *catA* gene in reference strain *P. mirabilis* HI4320 (**Sup Fig 3b**). The nucleotide and amino acid sequences of *catA1* from our isolates share 100% amino acid identity with *catA1* from *E. coli* and ~98% amino acid identity with *catA1* from *Photobacterium damselae* and *Shigella flexneri* (**Fig S3c)**. These findings indicate that the coexistence of multiple resistance genes can significantly alter phenotypic resistance, highlighting the complexity of chloramphenicol resistance in *P. mirabilis* clinical isolates.

## DISCUSSION

CAUTIs caused by *P. mirabilis* remain clinically challenging due to crystalline biofilm formation, AMR, recurrent infections, and persistence even after antibiotic treatment and catheter changes [15]. This comprehensive genomic analysis of *P. mirabilis* from human urinary sources expands our understanding of *P. mirabilis* genetic diversity, AMR profiles, and MLST prevalence. We studied the population structure and genomic diversity of a dataset of 1,001 publicly available genomes from human urinary sources supplemented by phylogenetic analysis of 27 clinical isolates, through which we determined that urinary *P. mirabilis* isolates possess an open pangenome with more than 41,121 gene families and a core genome of 1,937 genes for basic cellular functions, colonization, and survival.

In the context of bacterial genomics, open pangenomes are characteristics of sympatric species with a high capacity of horizontal gene transfer (HGT), highlighting genomic plasticity that may allow *P. mirabilis* to rapidly adapt to the urinary tract in the presence of antibiotic exposure, host immune responses, and competition in the polymicrobial catheterized environment [56-58]. *P. mirabilis* likely integrates genetic material from diverse sources, explaining the accessory genes that comprise the vast majority of the pangenome (37,918 genes, ~90%). This dynamic gain, loss, and exchange of genetic material emphasizes the importance of flexible genomic elements in driving diversity in the urinary tract.

The presence of 213 defined STs and approximately 118 previously undefined genomes and a star-like phylogeny further supports ongoing diversification. Almost half of the STs in the NCBI and clinical genomes were represented by single isolates (46%), showcasing heterogeneity. The majority of genomes were distributed across a small subset of STs (ST96, ST185, ST99, ST135, ST97), resulting in a skewed distribution. However, the highest AMR burdens were found in rarer STs (e.g., ST431, ST425, ST279). These strains carried numerous AMR gene subclasses, but we cannot comment if it is epidemiologically relevant due to low sampling (**Table 3**).

In this study we used MLST to identify lineages as it remains a widely adopted method of bacterial typing due to its reproducibility and ease of interpretation [49, 59]. However, for *P. mirabilis*, our results highlight limitations in the discriminatory power of MLST when applied alone for epidemiological and outbreak investigations [59, 60]. While MLST reliably identifies clonal expansions of prevalent lineages like ST96 and ST185, the large number of undefined STs in our analysis indicates gaps in current MLST schema and highlights the need for a continuously updated and standardized typing scheme, which we aim to do in the future.

Overall, the AMR landscape of the 1,001 urine genomes and 27 clinical isolates was complex and dynamic, with variable genotype-phenotype concordance. An important observation was the high prevalence of MDR, with 93% of the isolates resistant to ≥2 antibiotic subclasses and 25% resistant to >6 subclasses. This MDR burden poses a challenge for effective treatment of *P. mirabilis* urinary tract infections. The most important finding was the identification of similar antibiotic subclasses in same MLST even if they originated from different studies/geographical locations. We observed a lineage-specific AMR burden across STs. However, a widespread distribution of isolates with very high resistance gene counts (>20) across multiple STs indicate that extreme resistance gene accumulation is not restricted to ST but is a function of divergent genetic and environmental context. We can identify if a specific antibiotic subclass will be effective therapeutically by ST, at least in our top 15 isolates. However, this approach would not be useful for the >46% (**Fig 2a**) genomes with only 1-2 genomes per ST. Extending our analysis to more genomes for each rare ST will be necessary to verify the AMR-ST link of many *P. mirabilis* lineages.

The overwhelming prevalence of tetracycline resistance genes (98.9%), particularly *tetJ*, aligns with the well-documented intrinsic resistance of *P. mirabilis.* The dominance of *tetJ* and the frequent carriage of only a single *tet* gene (94.3%) suggest this determinant is often sufficient for the observed resistance phenotype. However, the prevalence of chloramphenicol resistance genes (*catA*, 95%) despite limited clinical use of chloramphenicol is striking. This phenomenon has been observed in other *Enterobacterales* and may be linked to co-selection with other antibiotics or acquisition due to extensive use in agricultural and environmental settings [61, 62]. *P. mirabilis* also exhibit the co-occurrence of *catA* and *catA1* in 19.2% of *cat*-positive isolates, with additional *floR* and *catB3* genes in a few isolates. Strains with only a single *cat* gene exhibited dose-dependent growth inhibition by chloramphenicol while the additional presence of *catA1* resulted in a hyper-resistant phenotype (no growth inhibition at all at any tested concentrations), enhancing the resistance phenotype. Detection of *catA1* in *P. mirabilis* is a significant finding as this gene has not been well reported and characterized in *P. mirabilis*. Since the sequences of both genes match those from closely related bacterial species, this may indicate HGT events via Mobile Genetic Elements (MGE) that carry *catA* [62] [63, 64].

Additionally, we observed resistance to important classes of antibiotics like aminoglycosides (71%) with common streptomycin resistance present within lineages that carried resistance determinants to other antibiotic classes as well, including carbapenems, cephalosporins, fluoroquinolones and trimethoprim-sulfonamides (**Fig 1**). The continued presence of resistance determinants for antibiotics such as aminoglycosides despite reduction in their clinical use may indicate either environmental influence or co-selection with other antibiotics in *P. mirabilis* urine isolates.

Trimethoprim (TMP, *dfr* genes, 29%) and sulfonamide (SMX, *sul* genes, 28%) resistance determinants were present at similar frequencies and co-occurred in 22% of genomes, which is likely due to the common use of TMP-SMX in UTI treatment and threatens the efficacy of this combination therapy for *P. mirabilis*. While ESBL-associated genes were less common (18.5% and primarily *blaTEM-1*), their presence is concerning for broader β-lactam resistance development. Resistance for quinolones (*qnr aac(6')-Ib-cr, gyrA/parC* mutations) and the chloramphenicol/florfenicol (*cat, floR*) combination were less common overall in both datasets (~7%) but worth noting because they act as molecular fingerprints of human driven selection under antimicrobial pressures due to their prevalence in agriculture [65-68].

Our comprehensive AMR analysis shows a complex landscape of genotype-to-phenotype relationships, which is characterized by both expected concordance and clinically critical discordance. The high degree of concordance observed for antibiotics like tetracycline was expected. Additionally, concordance observed for kanamycin, where presence of an *aph(3')-Ia* gene predicted a resistant phenotype, validated the robustness of the genomic approach for identifying known resistance determinants. It has been observed that enzymes like aminoglycoside-modifying enzyme like APH(3')-Ia confer high-level, constitutive resistance impacting clinical efficacy of these drugs independent of other mechanisms like efflux pumps [69-71]. Hence, we see a clear concordance where strains with *aph(3')-Ia* were resistant while the negative strains had sensitive MIC. Hence, the presence of AMR determinants are a good indication of resistance, but the lack of AMR determinants result in less clear resistance predictions.

Our phenotypic analysis also shows that genotyping alone cannot always predict resistance phenotypes and can indicate multiple mechanisms at play. The observed discordances provide compelling insights into the evolving and multifaceted nature of AMR. For example, resistance to streptomycin and streptothricin could be due to novel resistance mechanisms, such as alternate acetyltransferases with low sequence homology to known enzymes. Similarly, the ampicillin resistance in strains 102-24, 106-15, H94-00, H97-11, and HI4320 that lack a detectable β-lactamase gene could be due to non-enzymatic mechanisms or plasmids. In general, resistance mediated by permeability or efflux is a hallmark of concentration-dependent antibiotics, where higher antibiotic concentrations can eventually overcome cellular defenses in concentration-dependent antibiotics [72, 73]. This is an important consideration in clinical settings, as treating strains that exhibit dose-dependent resistance with a higher doses or adjuvant may still be effective, whereas strains with enzymatic resistances lacking dose-dependent inhibition may require a different approach altogether. Additionally, the genomic AMR prediction is limited by the existing databases for less well studied organisms like *P. mirabilis,* lowering the detection of AMR genes and mutations. Hence, this study highlights the need to characterize AMR mechanisms as well as update databases, given the clinical importance of this pathogen in UTI.

One of the most critical findings from our genotype-phenotype studies was the observation that genotypic susceptibility to SMX did not always correspond to phenotypic susceptibility to TMP-SMX combination treatment (**Fig. 8**). We identified a spectrum of strains that lacked SMX resistance factors that were either sensitive, resistant but inhibited in a dose-dependent manner, or completely resistant to combination treatment, suggesting the presence of unknown SMX resistance mechanisms that vary in their efficacy. This kind of prediction error can be very dangerous in a clinical setting since it could lead to treatment failure. Strains like 206-05 and 210-04 (with MIC >16/305 ug/mL) possess a mechanism that is robust enough to contribute to TMP-SMX resistance, albeit less efficiently than the truly resistant strains. In contrast, the sensitivity of strains like HI4320, H97-00, and 106-15 to the TMP-SMX combination treatment implies that the yet unidentified SMX resistance mechanisms can still be overcome by trimethoprim.

The chloramphenicol resistance profile further illustrates the nuanced relationship between genotype and phenotype. Isolates with a single *catA* gene exhibited a clear dose-dependent response, where increasing antibiotic concentrations led to progressively greater growth inhibition. In contrast, strains possessing both *catA* and *catA1* genes demonstrated a constitutive high-level resistance phenotype with robust growth maintained even at the highest concentrations tested. This may suggest a gene dosage effect, where combined expression of two acetyltransferases enhances resistance. Overall, these data demonstrate how genetic complexity within a strain can directly dictate the clinical level of resistance.

The genotype-phenotype discordance observed in our small sample study highlights a significant limitation of current genomic prediction models: their reliance on curated databases of known resistance genes and mutations. Our study confirms that WGS-based prediction is highly reliable for well-characterized resistance determinants in *P. mirabilis* but can fail to predict phenotypes that are likely governed by cryptic mutations or mechanisms involving efflux and permeability. Not much has been reported on the mechanism of resistance involving efflux systems and membrane permeability [10, 65, 74]. Efflux systems such as *AcrAB-TolC* are reported in many *P. mirabilis* strains  can be overwhelmed by high drug concentrations, rather than the efficient enzymatic hydrolysis provided by a beta-lactamase and is linked to a very high multidrug-resistance rate of□96□% [75].

Predictions based on genotype are also prone to sequencing and assembly/annotation errors and ideally need to be reinforced with long-read or reference-based sequencing to create hybrid assemblies. However, this can become impractical and costly for diagnostic laboratories. In conclusion, while WGS has the potential to be used widely in clinical context for AMR predictions, our findings reinforce that assessing gene presence/absence does not remove the necessity for phenotypic AST of *P. mirabilis* isolates for accurate clinical decision-making and surveillance. Further exploration of isolates that were predicted to be sensitive but were either fully resistant or exhibited dose-dependent growth inhibition may yield discovery of new resistance mechanisms in this problematic bacterial species.

## Acknowledgements

This work was supported by an American Heart Association (AHA) pre-doctoral fellowship 24PRE1195538 to ND, and by the National Institute of Diabetes and Digestive and Kidney Diseases under awards R01 DK123158 and R01 DK140371 to CEA.

**Table 1: Summary statistics of *P. mirabilis* genome sequences**

| **Publicly available *P. mirabilis* genomes isolated from urine** | | | | |
|---|---|---|---|---|
| **Metric** | **Average** | **Max** | **Min** | **Median** |
| **# Contigs** | 258.43 | 973 | 60 | 180 |
| **Total Length** | 4020761.51 | 8382181 | 3667799 | 3992140 |
| **N50** | 174294.28 | 1994055 | 4981 | 174571 |
| **GC Content** | 38.76 | 43.24 | 37.57 | 38.69 |
| | | | | |
| **Clinical *P. mirabilis* genomes isolated from catheterized individuals** | | | | |
| **Metric** | **Average** | **Max** | **Min** | **Median** |
| **# Contigs** | 66.52 | 98 | 2 | 69 |
| **Total Length** | 4015048.48 | 4165371 | 3795264 | 4015495 |
| **N50** | 317358.93 | 4063606 | 119871 | 182416 |
| **GC Content** | 38.74 | 38.98 | 38.38 | 38.78 |

**Table 2: *P. mirabilis* clinical isolates used in this study**

| Isolate ID | Study | Species | MLST | Patient designation in cited publication | Isolation week |
|---|---|---|---|---|---|
| 102-00 | CEA | *P. mirabilis* | 185 | Participant A [15] | Visit 0 |
| 102-24 | CEA | *P. mirabilis* | 145 | Participant A [15] | Visit 24 |
| 102-25 | CEA | *P. mirabilis* | UD | Participant A [15] | Visit 25 |
| 104-00 | CEA | *P. mirabilis* | UD | Participant C [15] | Visit 0 |
| 106-15 | CEA | *P. mirabilis* | 552 | Participant H [15] | Visit 15 |
| 201-07 | CEA | *P. mirabilis* | UD | Participant G [15] | Visit 7 |
| 203-01 | CEA | *P. mirabilis* | 615 | Participant F [15] | Visit 1 |
| 206-05 | CEA | *P. mirabilis* | 185 | Participant D [15] | Visit 5 |
| 206-16 | CEA | *P. mirabilis* | 185 | Participant D [15] | Visit 16 |
| 210-04 | CEA | *P. mirabilis* | 145 | Participant J [15] | Visit 4 |
| H81-01 | HUC | *P. mirabilis* | 765 | Patient 81-urine [17] | Visit 1 |
| H85-10 | HUC | *P. mirabilis* | 330 | Patient 85-urine [17] | Visit 10 |
| H94-00 | HUC | *P. mirabilis* | 696 | Patient 94-urine [17] | Visit 0 |
| H97-00 | HUC | *P. mirabilis* | 157 | Patient 97-urine [17] | Visit 0 |
| H97-11 | HUC | *P. mirabilis* | 157 | Patient 97-urine [17] | Visit 11 |
| H105-00 | HUC | *P. mirabilis* | 591 | Patient 105-urine [17] | Visit 0 |
| H105-06 | HUC | *P. mirabilis* | 765 | Patient 105-urine [17] | Visit 6 |
| H108-00 | HUC | *P. mirabilis* | 96 | Patient 108-urine [17] | Visit 0 |
| H115-00 | HUC | *P. mirabilis* | 135 | Patient 115-urine [17] | Visit 0 |
| H115-05 | HUC | *P. mirabilis* | 135 | Patient 115-urine [17] | Visit 5 |
| H119-03 | HUC | *P. mirabilis* | 146 | Patient 119-urine [17] | Visit 3 |
| H120-00 | HUC | *P. mirabilis* | 97 | Patient 120-urine [17] | Visit 0 |
| H120-04 | HUC | *P. mirabilis* | 185 | Patient 120-urine [17] | Visit 4 |
| H125-06 | HUC | *P. mirabilis* | 565 | Patient 125-urine [17] | Visit 6 |
| H127-00 | HUC | *P. mirabilis* | 96 | Patient 127-urine [17] | Visit 0 |
| H132-05 | HUC | *P. mirabilis* | 96 | Patient 132-urine [17] | Visit 5 |
| HI4320 | Reference | *P. mirabilis* | 90 | Refseq: GCA_000069965.1_ASM6996v1_genomic | - |

## Table 3: AMR subclass count and percentage in *P. mirabilis* genomes

| Resistance subclass | Count (NCBI) | Genes detected (NCBI) | Percentage (1001 NCBI) | Count (clinical) | Genes detected (clinical) | Percentage (26 clinical) | NCBI/Clinical Comparison |
|---|---|---|---|---|---|---|---|
| AMIKACIN/KANAMYCIN / QUINOLONE / TOBRAMYCIN | 11 | *aac(6')-Ib, aph(3')-IIIa, aph(3')-VI, aph(3')-VIb, aph(3')-XV* | 7.19% | 1 | *aac(6')-Ib-cr5* | 3.70% | Lower in clinical |
| BETA-LACTAM | 185 | *blaCARB-2, blaOXA, blaOXA-1033, blaOXA-2, blaOXA-9, blaTEM, blaTEM-1, blaTEM-2, blaTEM-30, hugA* | 18.46% | 5 | *blaTEM-1* | 18.52% | Similar in prevalence |
| CEPHALOSPORIN | 90 | *ampC, blaCMY-2, blaCMY-6, blaCTX-M-14, blaCTX-M-15, blaCTX-M-2, blaCTX-M-3, blaCTX-M-55, blaCTX-M-65, blaCTX-M-91, blaDHA, blaDHA-1, blaFOX-5, blaOXA-1, blaOXA-10, blaPDC-3, blaPER, blaPER-1, blaSHV-12, blaSHV-31, blaTEM-10, blaTEM-155, blaVEB-6* | 8.98% | 1 | *blaOXA-1* | 3.70% | Lower in clinical |
| CHLORAMPHENICOL | 891 | *catA, catA1, catA2, catA3, catB11, catB2, catB3, catB7, catB8, cmlA, cmlA5, cmlA6* | 88.92% | 23 | *catA, catA1, catB3, floR* | 85.19% | Similar in prevalence |
| ERYTHROMYCIN | 36 | *ere(A), ere(B) mph(E)* | 3.50% | 1 | *ere(A)* | 3.70% | Similar in prevalence |
| KANAMYCIN | 137 | *aph(3')-IIb, aph(3')-Ia* | 13.67% | 7 | *aph(3')-Ia* | 25.93% | Higher in clinical |
| QUATERNARY AMMONIUM | 192 | *qacE, qacEdelta1* | 19.16% | 3 | | 11.11% | Lower in clinical |
| QUINOLONE | 68 | *qnrA, qnrA1, qnrB19, qnrB2, qnrB4, qnrD, qnrD1, qnrD2, qnrS1, qnrVC1* | 6.79% | 2 | *qnrD* | 7.41% | Similar in prevalence |
| RIFAMYCIN | 28 | *arr-2, arr-3* | 2.79% | 1 | *arr-3* | 3.70% | Similar low prevalence |
| STREPTOMYCIN | 352 | *aadA1, aadA10, aadA16, aadA2, aadA3, aadA31, aadA5, ant(6)-Ia, aph(3'')-Ib, aph(6)-Id* | 35.13% | 10 | *aadA1, aph(6)-Id, aph(3'')-Ib, aadA5, aadA2* | 37.04% | Similar in prevalence |
| STREPTOTHRICIN | 282 | *sat2* | 28.14% | 10 | *sat2* | 37.04% | Higher in clinical |
| SULFONAMIDE | 286 | *sul1, sul2* | 28.54% | 6 | *sul1, sul2* | 22.22% | Slightly lower in clinical |

| | | | | | | | |
|---|---|---|---|---|---|---|---|
| TETRACYCLINE | 991 | *tet(59), tet(A), tet(B), tet(C), tet(D), tet(G), tet(H), tet(J), tet(L), tet(M), tet(Y)* | 98.90% | 26 | *tet(J), tet(A), tet(C)* | 100% | Nearly universal presence in both |
| TRIMETHOPRIM | 290 | *dfrA1, dfrA10, dfrA12, dfrA14, dfrA16, dfrA17, dfrA19, dfrA27, dfrA32, dfrA42, dfrA46, dfrA5, dfrA7* | 28.94% | 10 | *dfrA1, dfrA17, dfrA32* | 37.04% | Higher in clinical |
| AZITHROMYCIN / CLARITHROMYCIN / CLINDAMYCIN / ERYTHROMYCIN / TELITHROMYCIN | 1 | *erm(A)* | 0.10% | NA | NA | NA | Only in NCBI |
| AZITHROMYCIN / ERYTHROMYCIN / SPIRAMYCIN / TELITHROMYCIN | 15 | *mph(A)* | 1.5 | NA | NA | NA | Only in NCBI |
| AZITHROMYCIN / ERYTHROMYCIN / STREPTOGRAMIN | 26 | *msr(E)* | 2.59% | NA | NA | NA | Only in NCBI |
| BLEOMYCIN | 52 | *ble, bleO* | 5.19% | NA | NA | NA | Only in NCBI |
| CARBAPENEM | 171 | *blaIMP, blaIMP-27, blaKPC-2, blaKPC-3, blaNDM-1, blaNDM-4, blaNDM-5, blaNDM-50, blaNDM-6, blaNDM-7, blaOXA, blaOXA-23, blaOXA-48, blaVIM-1, blaVIM-78* | 17.07% | NA | NA | NA | Only in NCBI |
| FLORFENICOL | 61 | *floR, floR2* | 6.09% | NA | NA | NA | Only in NCBI |
| FOSFOMYCIN | 14 | *fosA, fosA3* | 1.40% | NA | NA | NA | Only in NCBI |
| GENTAMICIN | 54 | *aac(3)-IId, aac(3)-IIe, aac(3)-VIa, aac(6')-Ib, aac(6')-Ib4, armA* | 5.39% | NA | NA | NA | Only in NCBI |
| GENTAMICIN / KANAMYCIN / TOBRAMYCIN | 72 | *ant(2")-Ia* | 7.19% | NA | NA | NA | Only in NCBI |
| HYGROMYCIN | 13 | *aph(4)-Ia* | 1.30% | NA | NA | NA | Only in NCBI |
| KASUGAMYCIN | 1 | *aac(2')-IIa* | 0.10% | NA | NA | NA | Only in NCBI |
| LINCOSAMIDE | 42 | *lnu(F), lnu(G)* | 4.19% | NA | NA | NA | Only in NCBI |
| SPECTINOMYCIN / STREPTOMYCIN | 1 | *aadA27* | 0.10% | NA | NA | NA | Only in NCBI |

## Figure Legends

**Fig 1. Phylogenetic tree and pangenome analysis of publicly-available *Proteus mirabilis* genomes from human urine samples.** (**1a**) maximum-likelihood phylogenetic tree of 1,001 *P. mirabilis* human urine genomes with metadata arranged as concentric bands. The first two bands display multi-locus sequence type (ST), and the third band indicates the study source in the NCBI database associated with the Bioproject. Bands 4-8 indicate presence (colored bars) or absence (grey) of resistance genes across different antibiotic classes. Distinct clusters were observed that either had resistance genes for nearly all classes of antibiotic or lacked them entirely, which was dependent on ST and independent of geographical source. (**1b**) Roary collector curve showing conserved genes (~1,700 core genome) present in all of the genomes analyzed as well as the number of total genes, which increases as new genomes are added, indicating an open pangenome. (**1c**) Roary collector curve showing unique vs new gene accumulation across all *P. mirabilis* genomes. Here, the total unique genes is >15,000 indicating massive accessory gene diversity and an open pangenome. Early genomes introduce a lot of new genes before dropping out. However, after addition of >250 genomes, new genes (vertical lines) are still accumulated, indicating horizontal gene transfer (HGT), mobile genetic elements, or prophage insertions.

**Fig 2. Sequence type distribution of publicly-available *Proteus mirabilis* genomes from human urine samples.** (**2a**) Pie-chart illustrating the distribution of MLSTs grouped by their sample count frequency. (**2b**) Bar plot showing the top 20 MLSTs based on sample count.

**Fig 3: Phylogenetic tree and pangenome analysis of *Proteus mirabilis* strains isolated from individuals with indwelling urinary catheters.** (**3a**) A mid-rooted maximum likelihood phylogenetic tree of clinical *P. mirabilis* isolates collected from patients with long-term urinary catheters. The phylogenetic tree displays the participant ID, the cohort ID (strip1), and multi-locus sequence type (ST, strip 2). Many of the isolates cluster by ST, but not by geographical location. (**3b**). Mosaic pan genome with presence/absence matrix of core-genome phylogenetic tree for 26 *P. mirabilis* clinical isolates collected from patients with long-term urinary catheters.

**Fig 4: AMR gene carriage in publicly-available *P. mirabilis* genomes.** (**4a**) Number of *P. mirabilis* strains in the NCBI dataset carrying 1, 2, and 3 tetracycline resistance (*tet)* genes. (**4b**) Three-way Venn diagram exhibiting the shared chloramphenicol resistance genes (*catA, catA1,* and *floR*) by all the *P. mirabilis* isolates in the NCBI dataset. (**4c**) Number of *P. mirabilis* strains in the NCBI dataset carrying 1, 2, and 3 trimethoprim resistance (*dfrA)* genes. (**4d**) Venn diagram of shared sulfonamide resistance genes (*sul1* and *sul2*). (**4e**) Venn diagram of shared trimethoprim (TMP) and sulfonamide (SMX) resistance genes.

**Fig 5: Distribution of AMR subclass carriage in isolates from the most common STs.** (**5a**) AMR subclass count for the top MLSTs that each had >3 representative genomes. Each black point represents the AMR count for one isolate within the indicated ST. The overall dataset-wide subclass mean is indicated by the red line. (**5b**) Empirical cumulative distribution function (ECDF) showing the cumulative proportion of *P. mirabilis* genomes as a function of the number of AMR subclasses detected per genome, with $80^{th}$ percentile noted at 6 subclasses. (**5c**) Mean AMR subclass count for the 10 most prevalent multi-locus sequence types (ST), ordered by prevalence. Bars summarize the average burden of AMR subclasses per isolate in each ST. (**5d**) Distribution of AMR subclass count per isolate within each ST among the 10 most prevalent ST, with each black point representing an isolate. (**5e**) Empirical cumulative distribution function (ECDF) showing the cumulative proportion of isolates as a function of the number of AMR genes detected per isolate, with $80^{th}$ percentile noted at 18 genes.

**Fig 6: Overview of antibiotic resistance and MIC.** (**6a**) Overview of antibiotic resistance phenotype found in the clinical CAUTI isolates. Color coding denotes resistance status: red = resistant, yellow = intermediate resistance, and green = susceptible strains. The presence or absence of resistance-associated genes is indicated by filled and empty circles, respectively. (**6b**) Heatmap representing the MIC for each antibiotic tested across clinical *P. mirabilis* CAUTI isolates. Color intensity corresponds to MIC values, indicating variation in resistance levels.

**Fig 7: Dose response curves and MIC**: Mean normalized OD600 values at experimental endpoint (20 hours) are plotted against antibiotic concentrations in ug/ml.

OD is normalized to a no drug control set to 1.0 (maximum growth). The red vertical line indicates the resistance breakpoint (CLSI) for *Enterobacterales.* The MIC was determined as the lowest antibiotic concentration for which growth was restricted below a threshold of a normalized OD of 0.15 (grey horizontal dashed line). (**7a**) Tetracycline (MIC range tested: 2-20 ug/ml), (**7b**) Kanamycin (MIC range tested: 6-34 ug/ml), (**7c**) Streptomycin (MIC range tested: 4-22 ug/ml), (**7d**) Ampicillin (MIC range tested: 6-34 ug/ml), (**7e**) Streptothricin (MIC range tested: 4-22 ug/ml).

**Fig 8: Dose response curves and MIC**: Mean normalized OD600 values at experimental endpoint (20 hours) are plotted against antibiotic concentrations in ug/ml. OD is normalized to a no drug control set to 1.0 (maximum growth). The red vertical line indicates the resistance breakpoint (CLSI) for *Enterobacterales.* The MIC was determined as the lowest antibiotic concentration for which growth was restricted below a threshold of a normalized OD of 0.15 (grey horizontal dashed line). (**8a**) Sulfamethoxazole (MIC range tested: 250-512 ug/ml), (**8b**) Trimethoprim (MIC range tested: 4-22 ug/ml), (**8c**) TMP-SMX (MIC range tested: 0.5/9.5-16/305 ug/ml).

**Fig 9: Dose response curves and MIC of Chloramphenicol.** Mean normalized OD600 values at experimental endpoint (20 hours) are plotted against antibiotic concentrations in ug/ml. OD is normalized to a no drug control set to 1.0 (maximum growth). The red vertical line indicates the resistance breakpoint (CLSI) for *Enterobacterales.* The MIC was determined as the lowest antibiotic concentration for which growth was restricted below a threshold of a normalized OD of 0.15 (grey horizontal dashed line). Chloramphenicol concentrations tested ranged from 6-34 ug/ml.

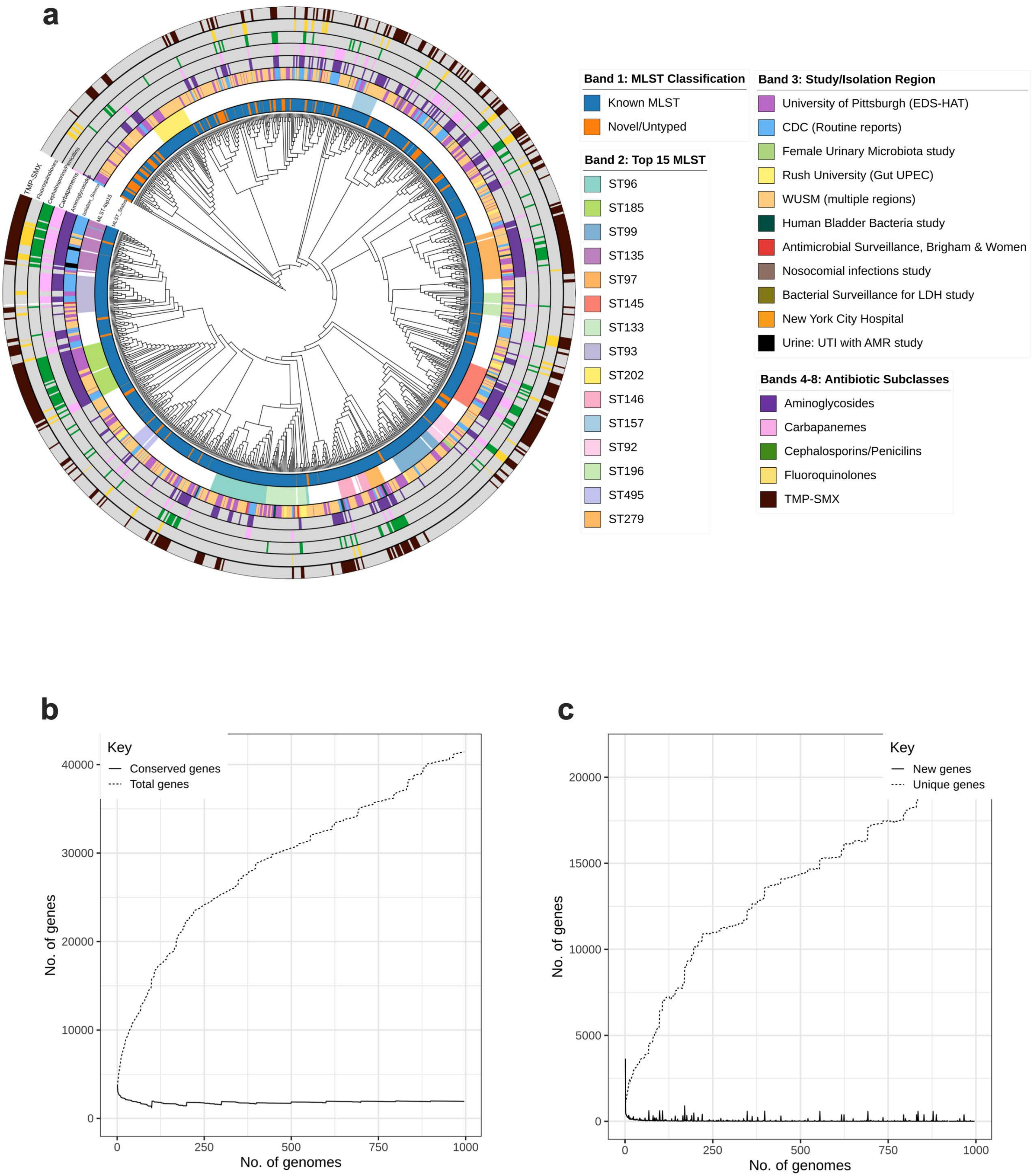

a
TMP-SMX
Fluoroquinolones
Cephalosporins/Penicilins
Carbapenems
Aminoglycosides
Isolation_Source
MLST-top15
MLST_Status
Band 1: MLST Classification
Known MLST
Novel/Untyped
Band 2: Top 15 MLST
ST96
ST185
ST99
ST135
ST97
ST145
ST133
ST93
ST202
ST146
ST157
ST92
ST196
ST495
ST279
Band 3: Study/Isolation Region
University of Pittsburgh (EDS-HAT)
CDC (Routine reports)
Female Urinary Microbiota study
Rush University (Gut UPEC)
WUSM (multiple regions)
Human Bladder Bacteria study
Antimicrobial Surveillance, Brigham & Women
Nosocomial infections study
Bacterial Surveillance for LDH study
New York City Hospital
Urine: UTI with AMR study
Bands 4-8: Antibiotic Subclasses
Aminoglycosides
Carbapenemes
Cephalosporins/Penicilins
Fluoroquinolones
TMP-SMX
b
Key
Conserved genes
Total genes
No. of genes
40000
30000
20000
10000
0
0
250
500
750
1000
No. of genomes
c
Key
New genes
Unique genes
No. of genes
20000
15000
10000
5000
0
0
250
500
750
1000
No. of genomes

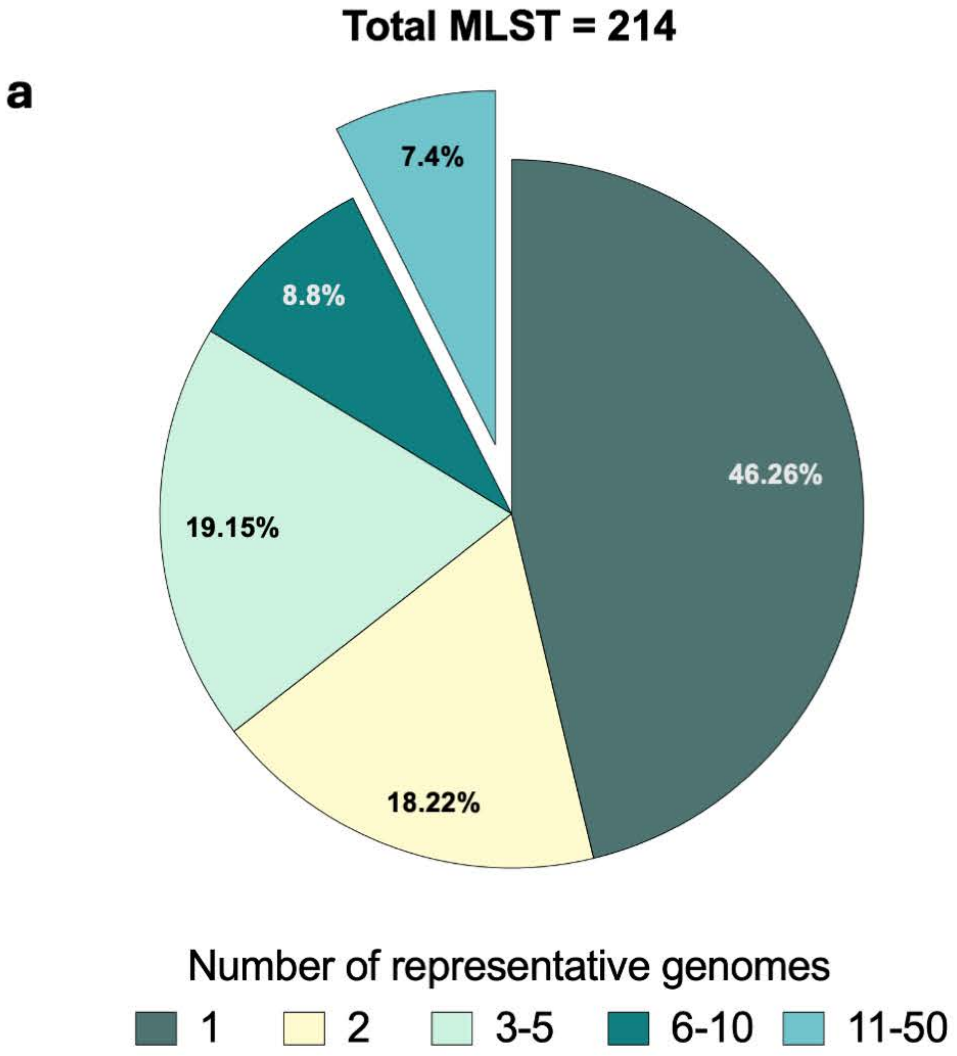

Total MLST = 214
a
7.4%
8.8%
46.26%
19.15%
18.22%
Number of representative genomes
1
2
3-5
6-10
11-50

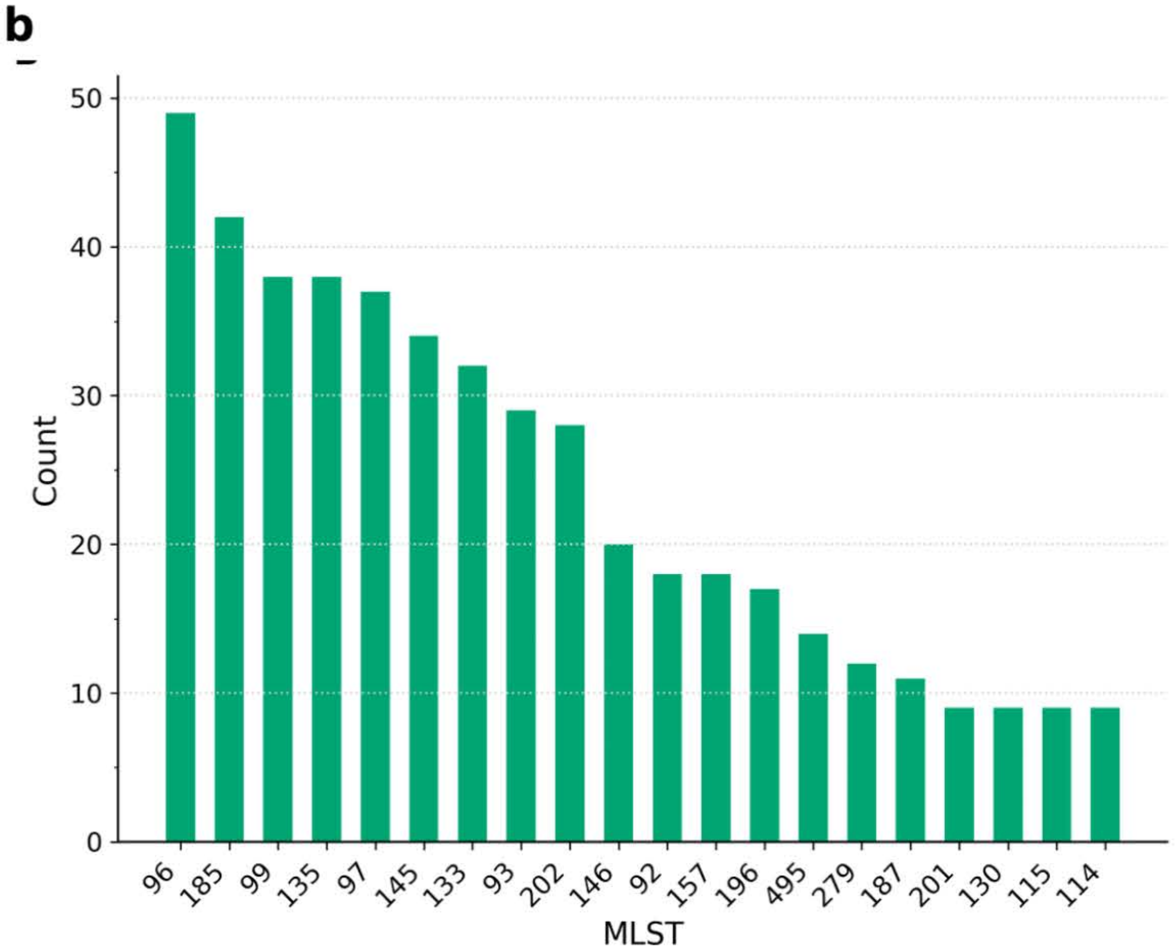

b
50
40
30
20
10
0
Count
96
185
99
135
97
145
133
93
202
146
92
157
196
495
279
187
201
130
115
114
MLST

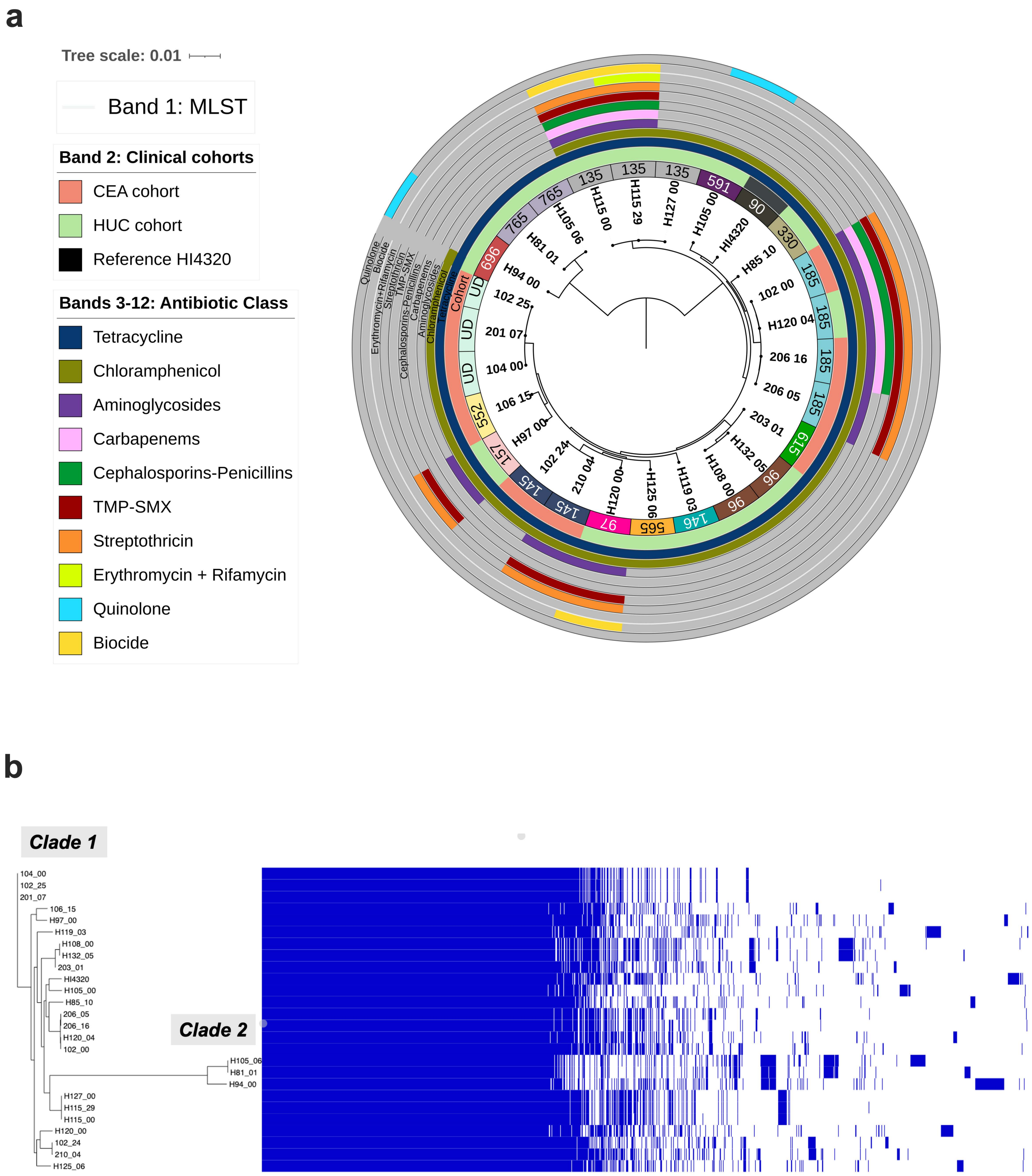

a
Tree scale: 0.01
Band 1: MLST
Band 2: Clinical cohorts
CEA cohort
HUC cohort
Reference HI4320
Bands 3-12: Antibiotic Class
Tetracycline
Chloramphenicol
Aminoglycosides
Carbapenems
Cephalosporins-Penicillins
TMP-SMX
Streptothricin
Erythromycin + Rifamycin
Quinolone
Biocide
b
Clade 1
Clade 2
104_00
102_25
201_07
106_15
H97_00
H119_03
H108_00
H132_05
203_01
HI4320
H105_00
H85_10
206_05
206_16
H120_04
102_00
H105_06
H81_01
H94_00
H127_00
H115_29
H115_00
H120_00
102_24
210_04
H125_06

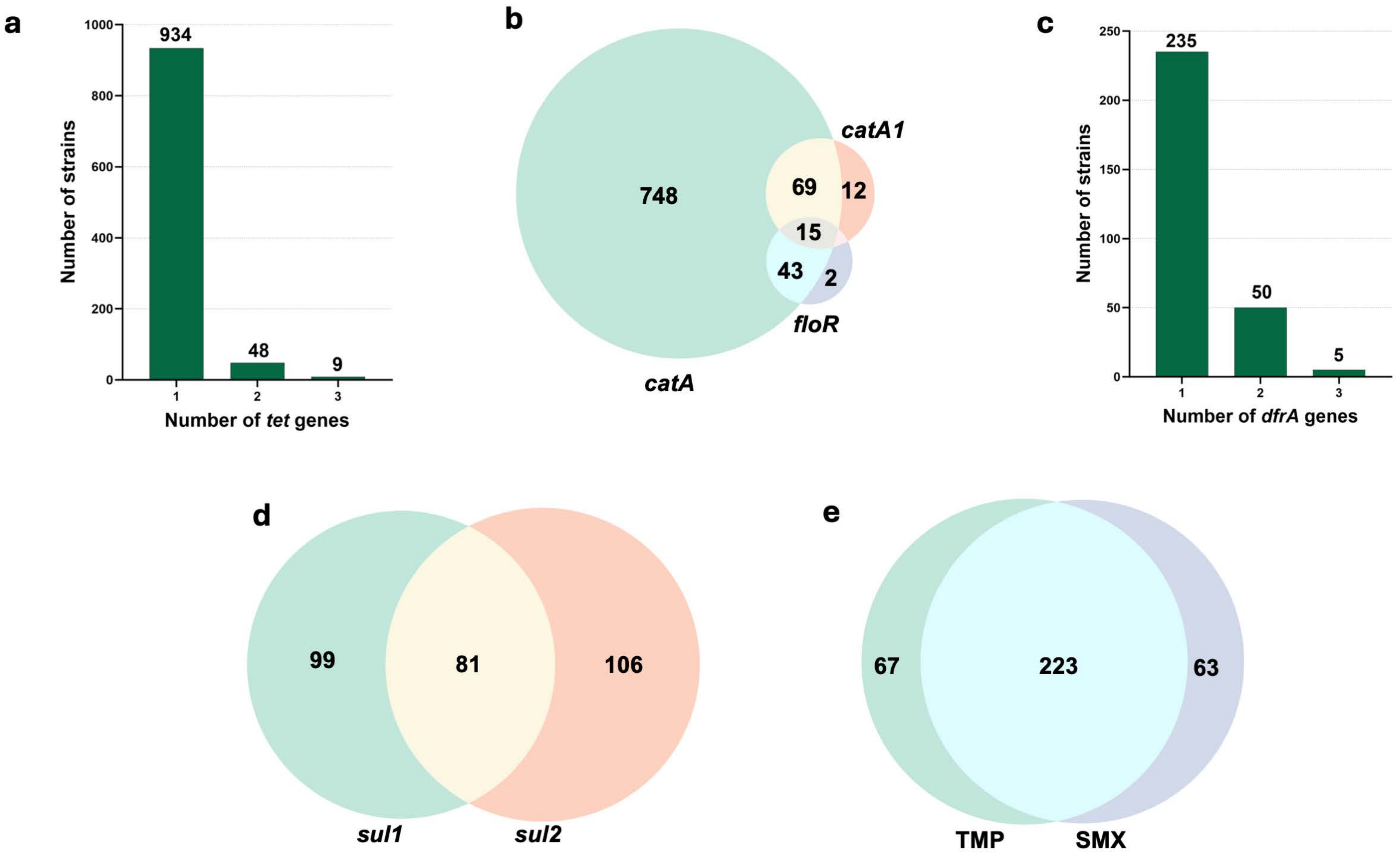

a
Number of strains
Number of tet genes
934
48
9
b
748
69
12
15
43
2
catA
catA1
floR
c
Number of strains
Number of dfrA genes
235
50
5
d
99
81
106
sul1
sul2
e
67
223
63
TMP
SMX

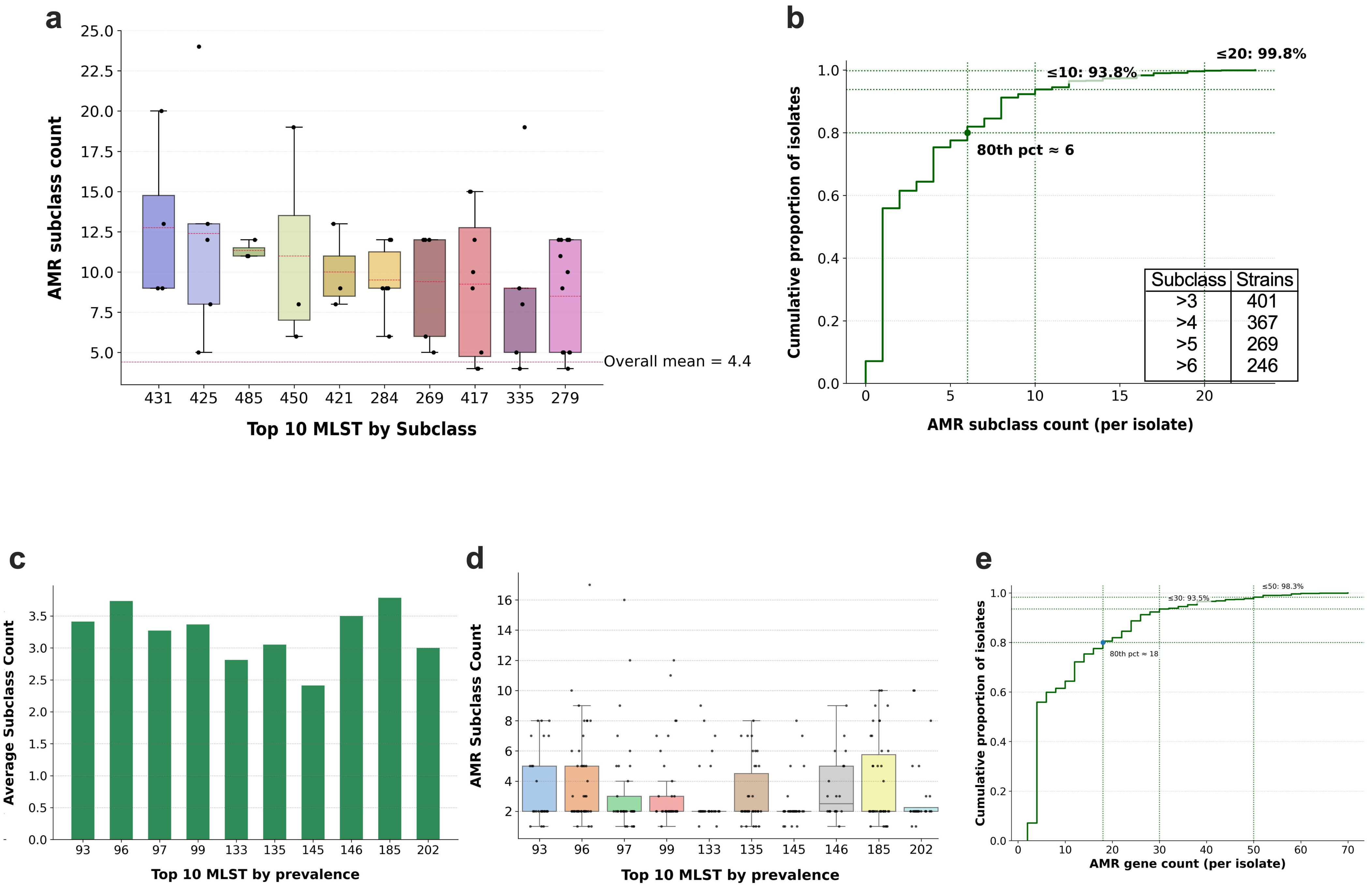

a
AMR subclass count
25.0
22.5
20.0
17.5
15.0
12.5
10.0
7.5
5.0
Overall mean = 4.4
431
425
485
450
421
284
269
417
335
279
Top 10 MLST by Subclass
b
Cumulative proportion of isolates
1.0
0.8
0.6
0.4
0.2
0.0
≤20: 99.8%
≤10: 93.8%
80th pct ≈ 6
Subclass | Strains
>3 | 401
>4 | 367
>5 | 269
>6 | 246
0
5
10
15
20
AMR subclass count (per isolate)
c
Average Subclass Count
3.5
3.0
2.5
2.0
1.5
1.0
0.5
0.0
93
96
97
99
133
135
145
146
185
202
Top 10 MLST by prevalence
d
AMR Subclass Count
16
14
12
10
8
6
4
2
93
96
97
99
133
135
145
146
185
202
Top 10 MLST by prevalence
e
Cumulative proportion of isolates
1.0
0.8
0.6
0.4
0.2
0.0
≤50: 98.3%
≤30: 93.5%
80th pct ≈ 18
0
10
20
30
40
50
60
70
AMR gene count (per isolate)

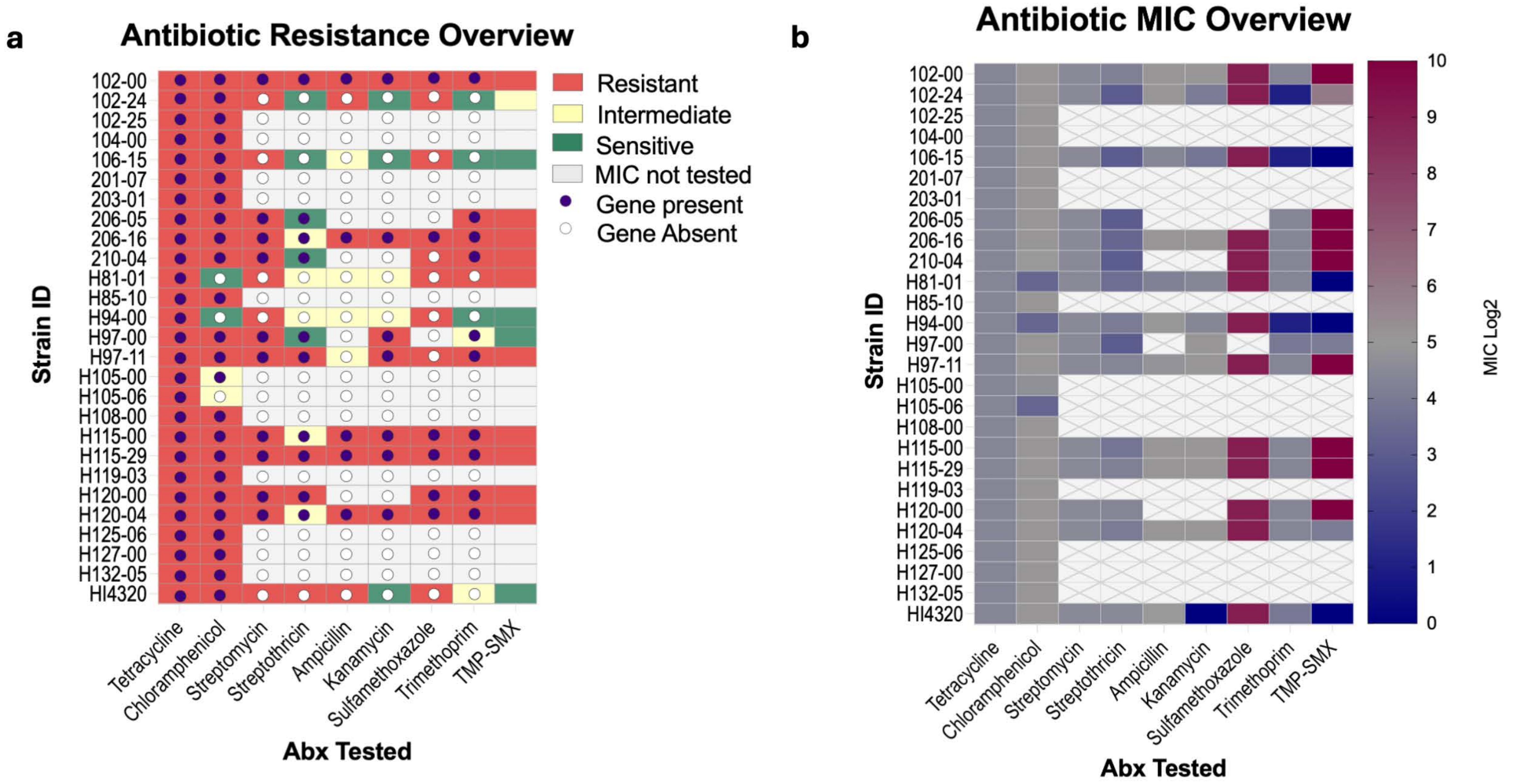

a
Antibiotic Resistance Overview
Resistant
Intermediate
Sensitive
MIC not tested
Gene present
Gene Absent
Strain ID
102-00
102-24
102-25
104-00
106-15
201-07
203-01
206-05
206-16
210-04
H81-01
H85-10
H94-00
H97-00
H97-11
H105-00
H105-06
H108-00
H115-00
H115-29
H119-03
H120-00
H120-04
H125-06
H127-00
H132-05
HI4320
Tetracycline
Chloramphenicol
Streptomycin
Streptothricin
Ampicillin
Kanamycin
Sulfamethoxazole
Trimethoprim
TMP-SMX
Abx Tested
b
Antibiotic MIC Overview
MIC Log2
0
1
2
3
4
5
6
7
8
9
10

a
Sulfamethoxazole dose-response
Normalized OD (endpoint mean)
Concentration (µg/mL)
102-00
102-24
206-16
106-15
H81-01
H94-00
H115-00
H115-29
H120-00
H120-04
HI4320
b
Trimethoprim dose-response
Normalized OD (endpoint mean)
Concentration (µg/mL)
102-00
102-24
106-15
206-05
206-16
210-04
H81-01
H94-00
H97-00
H115-00
H115-29
H120-00
H120-04
H97-11
HI4320
c
TMP-SMX dose-response
SMX Concentration (µg/mL)
9.5
19
38
76
152
305
Normalized OD (endpoint mean)
TMP- Concentration (µg/mL)
102-00
102-24
106-15
206-05
206-16
210-04
H81-01
H94-00
H97-00
H97-11
H115-00
H115-29
H120-00
H120-04
HI4320

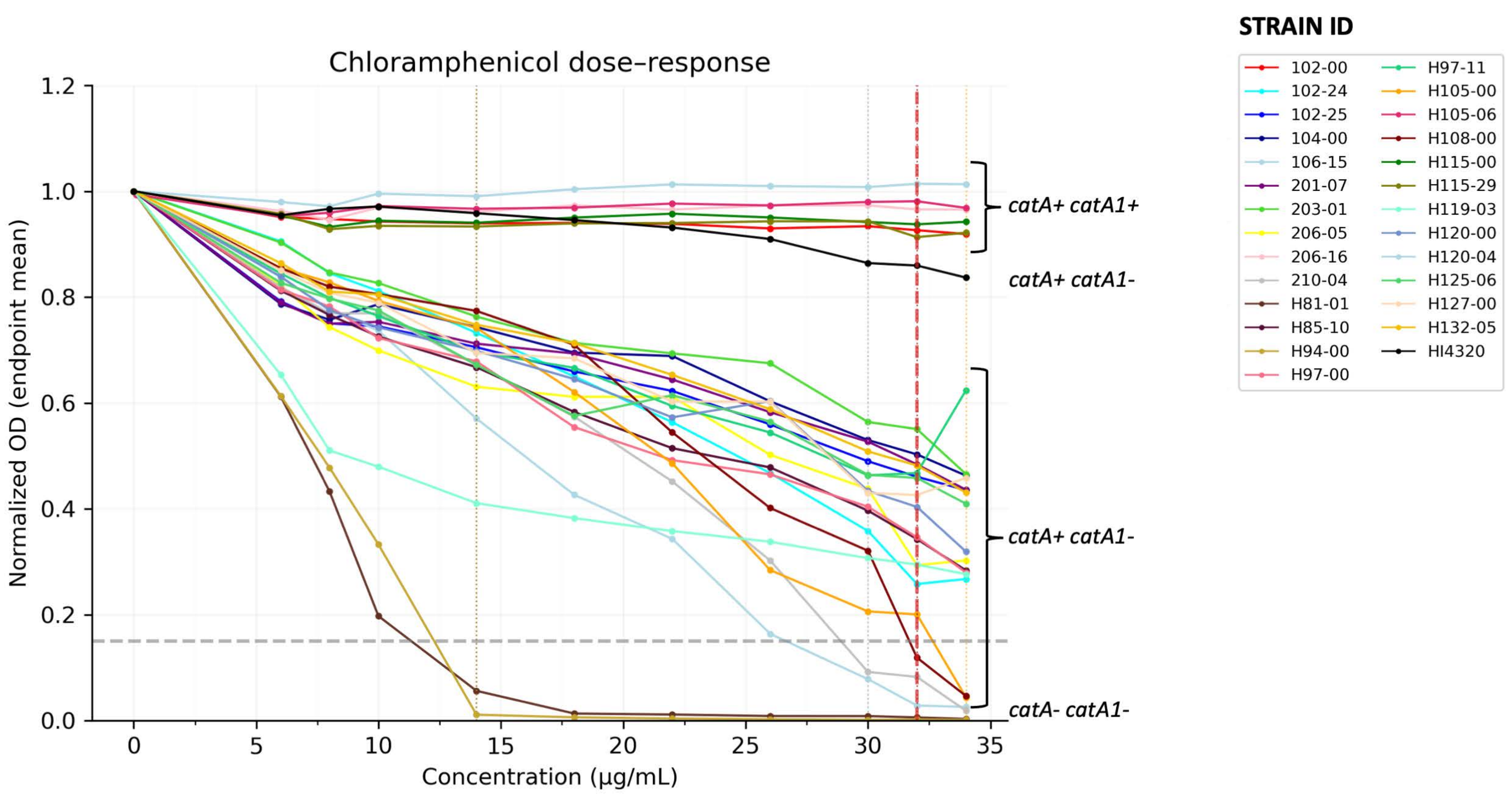

STRAIN ID
Chloramphenicol dose–response
Normalized OD (endpoint mean)
Concentration (µg/mL)
0.0
0.2
0.4
0.6
0.8
1.0
1.2
0
5
10
15
20
25
30
35
catA+ catA1+
catA+ catA1-
catA+ catA1-
catA- catA1-
102-00
102-24
102-25
104-00
106-15
201-07
203-01
206-05
206-16
210-04
H81-01
H85-10
H94-00
H97-00
H97-11
H105-00
H105-06
H108-00
H115-00
H115-29
H119-03
H120-00
H120-04
H125-06
H127-00
H132-05
HI4320
